# Self-assembled single-atom nanozyme for enhanced photodynamic therapy treatment of tumor

**DOI:** 10.1038/s41467-019-14199-7

**Published:** 2020-01-17

**Authors:** Dongdong Wang, Huihui Wu, Soo Zeng Fiona Phua, Guangbao Yang, Wei Qi Lim, Long Gu, Cheng Qian, Haibao Wang, Zhen Guo, Hongzhong Chen, Yanli Zhao

**Affiliations:** 1 Division of Chemistry and Biological Chemistry, School of Physical and Mathematical Sciences, Nanyang Technological University, 21 Nanyang Link, Singapore, 637371 Singapore; 2 Anhui Key Laboratory for Cellular Dynamics and Chemical Biology, School of Life Sciences, University of Science and Technology of China, Hefei, 230027 P. R. China; 3 Radiology Department of the First Affiliated Hospital of Anhui Medical University, Hefei, 230022 P. R. China; 4 School of Materials Science and Engineering, Nanyang Technological University, Singapore, 639798 Singapore

**Keywords:** Metal-organic frameworks, Self-assembly, Biomedical materials, Nanotechnology in cancer

## Abstract

Hypoxia of solid tumor compromises the therapeutic outcome of photodynamic therapy (PDT) that relies on localized O_2_ molecules to produce highly cytotoxic singlet oxygen (^1^O_2_) species. Herein, we present a safe and versatile self-assembled PDT nanoagent, i.e., OxgeMCC-r single-atom enzyme (SAE), consisting of single-atom ruthenium as the active catalytic site anchored in a metal-organic framework Mn_3_[Co(CN)_6_]_2_ with encapsulated chlorin e6 (Ce6), which serves as a catalase-like nanozyme for oxygen generation. Coordination-driven self-assembly of organic linkers and metal ions in the presence of a biocompatible polymer generates a nanoscale network that adaptively encapsulates Ce6. The resulted OxgeMCC-r SAE possesses well-defined morphology, uniform size distribution and high loading capacity. When conducting the in situ O_2_ generation through the reaction between endogenous H_2_O_2_ and single-atom Ru species of OxgeMCC-r SAE, the hypoxia in tumor microenvironment is relieved. Our study demonstrates a promising self-assembled nanozyme with highly efficient single-atom catalytic sites for cancer treatment.

## Abstract (web summary)

The hypoxic microenvironment in solid tumors limits the efficacy of photodynamic therapy (PDT) since oxygen is necessary to produce high cytotoxic singlet oxygen species. Here, the authors develop an improved self-assembled single-atom nanozyme which allows oxygen generation to enhance PDT efficacy.

## Introduction

Photodynamic therapy (PDT) has been selected as a clinical method for treating a wide range of superficial and localized cancer and other diseases since it utilizes excitation light, molecular oxygen (O_2_), and photosensitizer to generate high cytotoxic singlet oxygen (^1^O_2_) species^1–4^. Possessing temporal and spatial management over localization of the light irradiation, PDT could significantly enhance therapeutic efficiency and remarkably reduce side effect, thus showing superior advantages compared to conventional radiotherapy, chemotherapy, and surgery modalities^5–7^. However, PDT efficiencies are seriously compromised by solid tumor hypoxia due to the uncontrollable tumor growth and also the dysregulated formation of tumor blood vessels^8,9^. In addition, PDT also induce microvascular collapse which would block O_2_ transport and further aggravate the hypoxia tumor microenvironment. Consequently, this situation leads to a vicious cycle, as PDT induces tumor hypoxia, and tumor hypoxia in turn compromises PDT outcomes^10–12^. To solve this problem, one of the popular approaches is to design smart nanoplatforms for localized generation of O_2_ within tumor sites based on highly expressed intracellular H_2_O_2_ (5 nmol/10^5^ cells h^−1^)^13–15^. Various kinds of nanoplatforms such as MnO_2_, CaO_2_, carbon dots and biological catalase have been constructed to catalyze H_2_O_2_ into O_2_ for ameliorating tumor hypoxia^16–21^. While these nanoplatforms have shown the performance on alleviating the tumor hypoxia condition to some degree, low photosensitizer loading capacity (usually below 5 wt%) resulted in high dose usage of nanoplatforms has precluded their further clinical applications. Furthermore, rapid pH-responsive degradation of MnO_2_ that was thought to be advantageous because of its application for magnetic resonance (MR) imaging may be a shortcoming, since this translates to short-lived O_2_-generation^22^. Thus, developing nanoplatforms to provide a continuous O_2_ supply for long-term hypoxia amelioration is still a challenging task yet to be solved. There is a critical need for construction of more biocompatible PDT platforms with the ability to catalyze O_2_ generation continuously without self-consumption.

Recently, single-atom nanocatalysts with isolated active metal centers anchored on solid supports presented new breakthroughs in cost-effective catalysis^23,24^. The well-defined atom level dispersion could promote the atom economy for the metal usage through maximizing the atom utilization efficiency. Moreover, unique structure and coordination environment endow these materials with enhanced catalytic activity in many reactions with superior stability^25–27^. Therefore, the construction of single-atom catalysts is a powerful strategy for organismic biochemical reactions due to the highest atom utilization and abundant active sites offered. For instance, Liang and coworkers reported single-atom gold anchored carbon dots as a mitochondrial reactive oxygen species (ROS) amplifier for enhanced cancer treatment^28^. Xu et al. explored a single-atom zinc-based nanozyme for wound antibacterial applications^29^. Thus, developing optimal supporting materials with high catalyst loading, good biocompatibility, and low toxicity has still been sought after by scientists. In preference to conventional catalyst supports, nanoscale metal–organic frameworks (MOFs), constituted by metal nodes/ions and organic bridging linkers, have emerged as promising supports on account of their well-defined coordination network and tunable pore size^30–34^. The possibility of constructing single-atom nanocatalysts using MOFs as supporting materials was explored. Farha and coworkers reported MOF-supported single-metal-atom vanadium species with superior catalytic activities toward 4-methoxybenzyl alcohol^35^. As a subclass of MOFs, Prussian blue analogues (PBAs) with distinctive cubic M_a_[M_b_(CN)_6_] skeleton have been thoroughly studied because of their facile and mild synthesis as well as intrinsic multifunctional properties. Mn_3_[Co(CN)_6_]_2_ (MC), a type of PBAs, with an established double-perovskite framework where Mn is coordinated to six neighboring nitrogen exhibits excellent MR imaging ability due to high-spin Mn–N_6_ (S = 5/2) octahedra structure in the skeleton^36^. Many studies demonstrated that noble metal elements such as Pt, Pd and Ir could partially substitute the M_a_ or M_b_ position without perturbing the M_a_[M_b_(CN)_6_] framework for the construction of high performance electrochemical catalysts^37–39^. It was also reported that noble metal nanoparticles such as Ru, Pt and Au nanoparticles have catalase-like nanozyme activities^40–42^. In addition, the polarizable *π*-electron cloud of [Co(CN)_6_] may have a certain affinity to cargos with conjugated structures^43^. Thus, during organic linker-metal coordination process, various bioactive molecules could be conveniently encapsulated, indicating that the metal-organic coordination might be an efficient approach for incorporating single-atom catalytic active sites and therapeutic agents in the assembled materials.

**Fig. 1:**
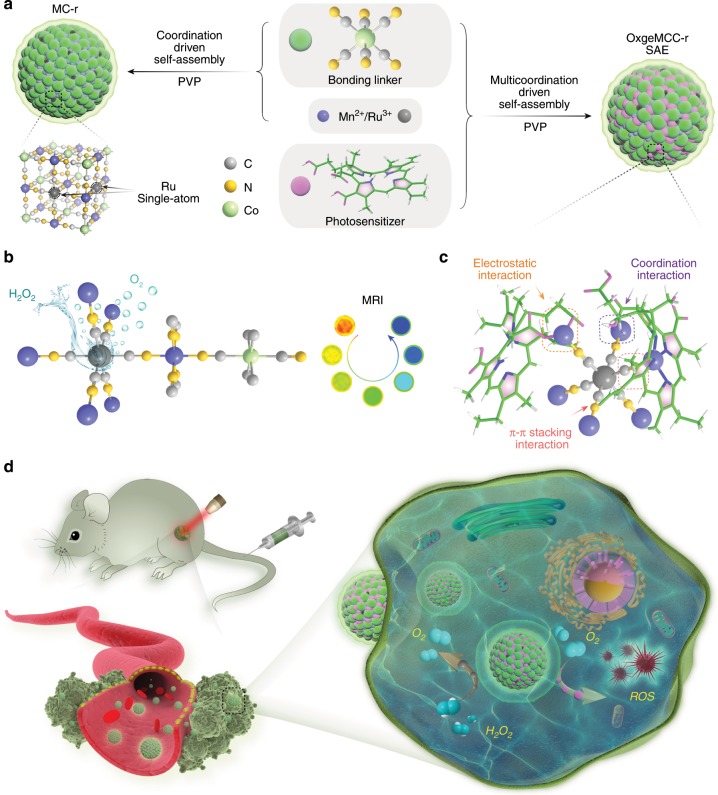
Design of OxgeMCC-r nanozyme with single-atom Ru for cancer treatment. **a** Schematic illustration of OxgeMCC-r. OxgeMCC-r consists of catalytically active single-atom Ru site anchored in MCC with outer PVP protection layer. **b** Partial molecular structure of OxgeMCC-r with active single-atom Ru site serving as catalase-like nanozyme for oxygen generation. **c** Multicomponent coordination interactions within the OxgeMCC-r SAE. **d** Scheme of continuously catalytic oxygen generation and ROS production for enhanced PDT of cancer by OxgeMCC-r SAE.

In this work, by using Mn_3_[Co(CN)_6_]_2_ MOF as the support material, we incorporate single-atom Ru into the framework with the loading weight ratio of up to 2.23 wt%, where Ru partially substitutes Co to serve as single-atom catalytic site for endogenous oxygen generation. Facilitated by collective coordination and other noncovalent interactions (Fig. 1), organic ligand, metal ions, and chlorin e6 (Ce6) photosensitizer encapsulated by biocompatible poly-vinylpyrrolidone (PVP) polymer could self-assemble to produce the well-defined and uniform single-atom enzyme (OxgeMCC-r SAE). Some of the advantages of OxgenMCC-r SAE include high Ce6 loading capacity due to intrinsic porous property of the MOF, high catalytic ability, and high catalytic durability for rapid O_2_ generation from endogenous H_2_O_2_ without being self-consumed or requiring external activation. The high catalytic activity of this nanozyme should be attributed to six unsaturated Ru–C_6_ coordination sites, leading to rapid decomposition of H_2_O_2_ with atomic economy to overcome the tumor hypoxia. T_1_-weighted MR imaging is also achieved using the resultant OxgeMCC-r SAEs on account of the existence of high-spin Mn–N_6_ (S = 5/2) species, which permits in vivo tracking of the therapeutic agent.

## Results

### Preparation and characterization

**Fig. 2:**
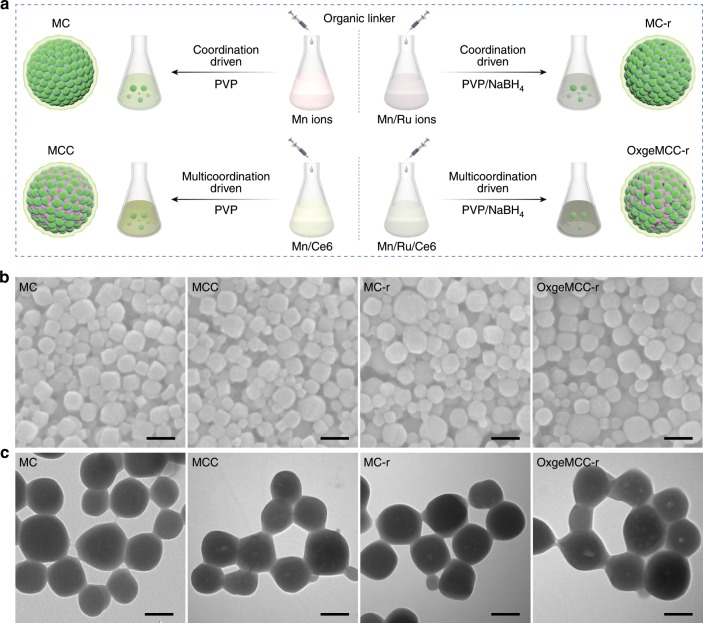
Synthesis and morphology characterizations. **a** Schematic illustration for the self-assembly of MC, MCC, MC-r nanoparticles, and OxgeMCC-r SAE. The organic linker [Co(C≡N)_6_] in syringe was added dropwise into four conical flasks filled with different systems under vigorous stirring. Turbidity was observed immediately upon the addition of [Co(C≡N)_6_] in the presence of PVP surfactant. **b** SEM images of the as-prepared MC, MCC, MC-r, and OxgeMCC-r. Scale bar is 100 nm. **c** Corresponding TEM images. Scale bar is 50 nm.

As illustrated in Fig. 1, the process for in situ one-pot multicomponent self-assembly of OxgeMCC-r SAEs involves collective coordination, hydrophobic, and electrostatic interactions among organic linker, PVP polymer, photosensitizer, and metal ions. A milk-white colloidal suspension generated immediately upon adding solution of organic [Co(C≡N)_6_] bridging ligand (1.54 mg mL^−1^) into solution of Mn^2+^ (0.59 mg mL^−1^) in the presence of surfactant PVP, indicating the formation of Mn_3_[Co(CN)_6_]_2_ nanoparticles (designated as MC) (Fig. 2a)^44^. Scanning electron microscopy (SEM) and transmission electron microscopy (TEM) results showed nearly spherical MC nanoparticles with an average size distribution of ~80 nm (Fig. 2b, c). Upon the addition of Ru^3+^ (0.072 mg mL^−1^) to the initial solution, partial substitution of Co was achieved due to stronger coordination ability between the terminal carbon of the ligand and Ru as compared with Co^39^. Essentially, Ru was doped in the framework via a post-exchange reaction^37^. Then, the doped Ru nodes were reduced under a mild reduction environment with low concentration NaBH_4_ as a reducing agent. The obtained Mn_3_[Co(CN)_6_]_2_-Ru (MC-r) nanoparticles also showed an uniform size of about 80 nm with darker TEM contrast on account of the existence of high-Z Ru atom. Herein, Ce6 was chosen as a hydrophobic photosensitizer, which could be incorporated within the MC or MC-r nanoparticles during the multicomponent self-assembly process. Adding an aqueous solution of organic [Co(C≡N)_6_] linker into a mixed solution of Ce6 (0.86 mg mL^−1^) and Mn^2+^ yielded dark-green MC-Ce6 (named as MCC), while the addition of Ce6 into a mixed aqueous solution of Mn^2+^ and Ru^3+^ afforded black-green OxgeMCC-r SAEs. SEM and TEM images also revealed that MCC and OxgeMCC-r were crack-free nanoparticles with the diameter of about 80 nm.

**Fig. 3:**
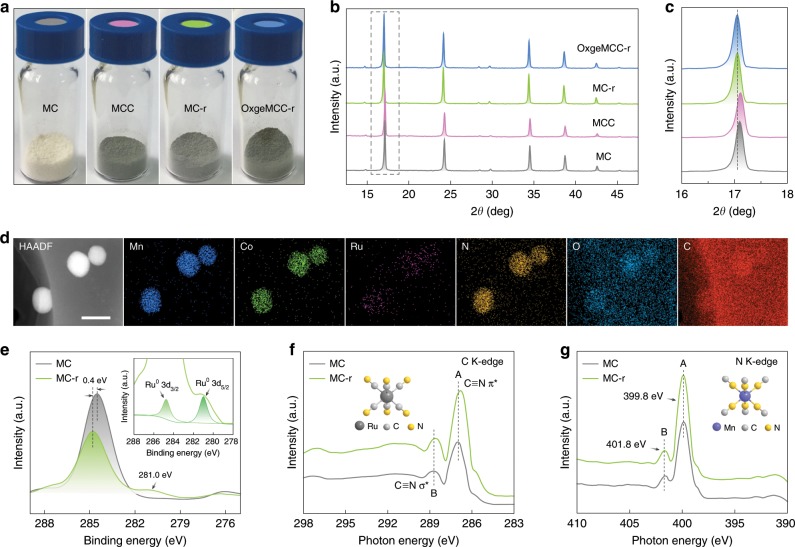
Structure characterizations. **a** Photograph of MC, MCC, MC-r, and OxgeMCC-r SAE. **b** Powder XRD of corresponding materials. **c** Enlarged powder XRD (2θ from 16 to 18 degree) of **b**. **d** HAADF-STEM image and corresponding EDS elemental mapping (Mn, Co, Ru, N, O, and C elements) of OxgeMCC-r SAEs. Scale bar is 100 nm. **e** C 1*s* XPS spectrum magnified from Supplementary Figs. 1 and 3. Inset is the enlarged Ru 3*d* spectrum of MC-r. **f** C K-edge NEXAFS spectra of MC and MC-r. **g** N K-edge NEXAFS spectra of MC and MC-r.

The color of the MCC, MC-r, and OxgeMCC-r powder changing from light-green to dark-green and then to black-green also confirmed the stepwise incorporation of Ce6 and the Ru substitution (Fig. 3a). Powder X-ray diffraction (PXRD) patterns showed that the obtained MC, MCC, MC-r, and OxgeMCC-r were of high crystallinity with sharp diffraction peaks (Fig. 3b). The appearance of major XRD peaks suggests that MC maintains its original framework structure during the Ru doping process, which is consistent with the unchanged morphology after the substitution reaction. Moreover, a slight shift to lower angle of the peak position centered at 2θ ~ 17^o^ was observed for MC-r and OxgeMCC-r as compared with that of MC and MCC, respectively (Fig. 3c). This phenomenon also indicates that some Co sites are substituted by bigger Ru during the self-assembly process. In addition, no additional diffraction peak suggests the absence of crystal Ce6 or Ru-based species in the OxgeMCC-r SAEs. These isolated Ru ions in the framework could serve as active catalytic sites for the decomposition of endogenous H_2_O_2_ toward the generation of O_2_. High-angle annular dark-field scanning transmission electron microscopy (HAADF-STEM) image and the corresponding energy-dispersive X-ray spectroscopy (EDS) elemental mapping (Fig. 3d) further confirm the homogeneous distribution of Mn, Co, Ru, N, O, and C elements throughout the OxgeMCC-r SAEs with single-atom Ru content of 2.18 wt%, consistent with the result based on inductively coupled plasma (ICP) measurement (Supplementary Table 1).

X-ray photoelectron spectroscopy (XPS) of MC and MC-r was also performed to identify the existing form of different species. Obviously, Ru 3*p* peaks at 462.1 and 485.3 eV were assigned to Ru 3*p*_3/2_ and Ru 3*p*_1/2_, respectively (Supplementary Figure 1). As shown in Fig. 3e, an obvious position change for the C 1 *s* peak from 284 to 284.4 eV was observed after the Ru doping, confirming that Ru took the place of some Co sites^39^. A Ru 3*d* XPS signal was observed but obscured by the C 1 *s* signal at 284.4 eV (inset of Fig. 3e). The deconvoluted spectrum presents one doublet (Ru 3*d*_3/2_ at 285.1 eV and Ru 3*d*_5/2_ at 281.0 eV), denoting a main valence state of Ru^0^ species, which is obviously different from that of pure RuCl_3_ powder (Supplementary Fig. 2)^45^. To investigate the electronic structure, near-edge X-ray absorption fine structure (NEXAFS) measurements were then performed. For the C K-edge spectrum of MC (Fig. 3f), peaks A (286.8 eV) and B (288.6 eV) are assigned to the excitations of C≡N π^*^ and C≡N σ^*^^46,47^. As compared with MC, the positions of peaks A and B in MC-r showed slight shifts to lower energy, indicating the changed 2*p*-band electron density of carbon. This phenomenon is originated from the electron donation from electron-rich Ru single atom to the nearest six carbons, whose *p*-electrons synergistically facilitate the stabilization of Ru single atom. Furthermore, unchanged positions from N K-edge spectrum centered at 399.8 eV (peak A) and 401.8 eV (peak B), together with Mn 2*p* for MC and MC-r, reveal that the N_6_ coordinative environment is unchanged (Fig. 3g and Supplementary Figure 3). Thus, the formation of single-atom Ru could be simply achieved by the reduction of these noble metal nodes in the presence of low concentration NaBH_4_ in the solution^48,49^.

**Fig. 4:**
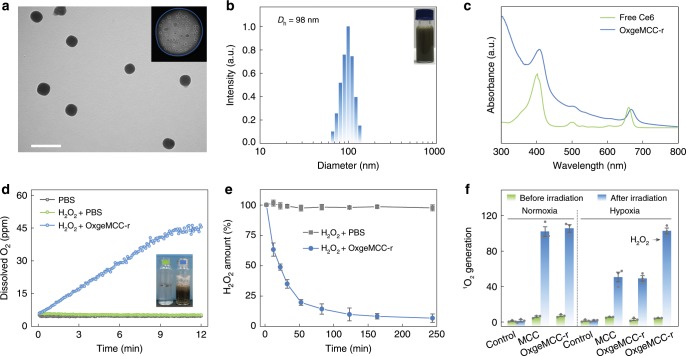
Structure, oxygen generation, and singlet oxygen generation of OxgeMCC-r SAE. **a** Representative TEM image. Inset is the enlarged image of one single OxgeMCC-r SAE after reversed-phase treatment. White dotted circles indicate the encapsulated Ce6. Scale bar is 200 nm. **b** DLS profile with the inset picture of the sample dispersed in water. **c** UV-vis absorption spectra of free Ce6 and OxgeMCC-r SAE. **d** O_2_ generation after treating OxgeMCC-r SAE with H_2_O_2_ in PBS. Inset is a photograph of H_2_O_2_ solutions in the presence or absence of OxgeMCC-r SAE. **e** Degradation profiles of H_2_O_2_ with or without of OxgeMCC-r SAE. **f** Singlet oxygen (^1^O_2_) generation ability determined by DPBF indicator under different conditions before and after laser irradiation (671 nm, 100 mW cm^−2^, 30 s). Data are presented as mean ± s.e.m. (*n* = 3).

As shown in Fig. 4a, the obtained OxgeMCC-r SAEs showed a well monodispersed near-globular morphology with a uniform PVP shell. It should be noted that PVP is a synthetic polymer with good biocompatibility to enhance the stability of OxgeMCC-r SAEs in physiological environment^50,51^. The dark region circled with white indicated that Ce6 was successfully incorporated into the self-assembled system (inset of Fig. 4a). Dynamic light scattering (DLS) measurements suggested relatively larger hydrophilic diameter as compared with the TEM image on account of the PVP coating (Fig. 4b). There was no apparent change in the DLS size within 12 days of storage, and the aqueous dispersion of OxgeMCC-r SAEs remained clear and stable (Supplementary Figure 4). By adjusting the amount of added Ce6 from 0 to 120 mg, corresponding loading capacity and loading efficiency varied, and an optimal Ce6 amount of 60 mg was chosen for the following experiments (Supplementary Figure 5). The UV-vis-NIR spectrum confirmed the successful incorporation of Ce6 with high loading capacity of 30.3 wt% and loading efficiency of 75.8% (Fig. 4c). The red-shift of the main absorbance peaks for Ce6 in the self-assembled system indicated the interaction between Mn and COO^−^ group as well as the *π*-*π* stacking interaction between Ce6 and organic linker. Furthermore, N_2_ adsorption and desorption isotherms were conducted to study the encapsulation of Ce6. As shown in Supplementary Fig. 6, both Brunauer-Emmett-Teller surface area (176.5 m^2^ g^−1^) and pore volume (0.12 cm^3^ g^−1^) of OxgeMCC-r SAEs were significantly lower than that of MC-r (735.8 m^2^ g^−1^ and 0.39 cm^3^ g^−1^), indicating the occupancy of pores by Ce6 in OxgeMCC-r.

### Long-term catalytic ability

After successful synthesis and morphological characterization of OxgeMCC-r SAE, we investigated its capability as a catalyst for converting H_2_O_2_ to O_2_. In the presence of H_2_O_2_ (10 mM), the O_2_ generation in the working solution gradually increased upon the addition of MC-r and OxgeMCC-r (Fig. 4d and Supplementary Fig. 7). Conversely, the control group without MC-r and OxgeMCC-r showed no change within the test period. High catalytic ability would reduce the catalyst dosage and promote its further biological applications. The experimental results indicated that, in the presence of MC-r and OxgeMCC-r, 50% of the added H_2_O_2_ could be consumed within 20 min (Fig. 4e and Supplementary Fig. 8). In addition, the catalytic reaction rate constant of OxgeMCC-r was calculated to be 0.041 min^−1^, which was higher than that of widely used MnO_2_ (0.029 min^−1^) under the same active component concentration (0.5 ppm Ru for OxgeMCC-r and 0.5 ppm Mn for MnO_2_, Supplementary Fig. 9). Thus, the anchored single-atom Ru catalytic site within the self-assembled system would be a superior catalyst toward H_2_O_2_, showing even better catalytic activity than the well-known MnO_2_^52^. Furthermore, the catalytic activity of OxgeMCC-r remained unchanged in a mimetic acidic tumor microenvironment (pH = 6.5) after several repeated additions of H_2_O_2_, suggesting its durable catalytic stability (Supplementary Fig. 10). On the other hand, catalytic ability of MnO_2_ rapidly decreased after two rounds of tests due to acid-induced catalyst self-decomposition (Supplementary Fig. 11). Since achieving continuous O_2_ supply for long-term hypoxia amelioration is a big challenge that hampers the PDT efficacy in tumor, the as-prepared OxgeMCC-r SAEs offer a promising solution to address this issue. Following which, the capability of OxgeMCC-r SAEs for enhanced PDT under a mimetic H_2_O_2_ environment was investigated. The ^1^O_2_ generation efficiency was measured with 1,3-diphenylisobenzonfuran (DPBF) as ^1^O_2_ indicator. As shown in Fig. 4f, in hypoxia condition, the ^1^O_2_ production ability of OxgeMCC-r SAEs under 671 nm laser irradiation was hampered compared to that in normoxia condition. Interestingly, with addition of H_2_O_2_, a comparable ^1^O_2_ production was achieved under both hypoxia and normoxia conditions in the presence of OxgeMCC-r SAEs. Considering the mimetic H_2_O_2_ environment and hypoxia condition, the improved ^1^O_2_ production could be attributed to the amelioration of hypoxic atmosphere through the single-atom based catalysis.

### Intracellular oxygen generation

**Fig. 5:**
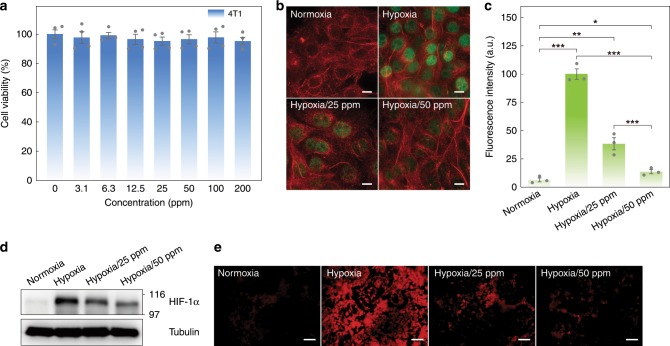
In vitro biocompatibility, alleviation of hypoxic condition, and intracellular O_2_ generation. **a** 4T1 cells viability after treated with MC-r nanoparticles with different concentrations. Data are presented as mean ± s.e.m. (*n* = 4). **b** Fluorescence imaging of 4T1 cells with stained HIF-1α (green) and tubulin (red) after treated with PBS under normoxic condition (21% O_2_, 5% CO_2_, and 74% N_2_) as well as PBS (Hypoxia) and MC-r (Hypoxia/25 ppm and Hypoxia/50 ppm) under hypoxic condition (1% O_2_, 5% CO_2_, and 94% N_2_). Scale bar is 10 µm. **c** Relative intensity of corresponding green fluorescence from HIF-1α under different treating conditions. Data are presented as mean ± s.e.m. (*n* = 3). Statistical analysis was performed via one-way ANOVA. *p < 0.05, **p < 0.01, ***p < 0.001. **d** Western blot analysis of HIF-1α expression in 4T1 cells under different treating conditions. **e** Fluorescence imaging of 4T1 cells stained by O_2_ indicator after treatments with different conditions. Scale bar is 100 µm.

On the basis of above results, endogenous H_2_O_2_ decomposition and intracellular oxygen generation of MC-r were investigated subsequently. The cytotoxicity was first studied before conducting further biological applications. More than 88% of 4T1 cells survived when incubated with MC-r at a concentration as high as 200 ppm (Fig. 5a), indicating satisfactory biocompatibility of the obtained MC-r nanoparticles. It is well established that hypoxia condition induces intracellular expression of HIF-1α protein^53^. We then wanted to understand how the level of HIF-1α in hypoxic cells changes after incubation with MC-r. In a typical experiment, HIF-1α protein and tubulin were stained using anti-HIF-1α antibody and anti-α-Tubulin antibody, respectively. As shown in Fig. 5b, the immunofluorescence imaging results showed no obvious expression of HIF-1α under normoxia condition. In contrast, when incubated under hypoxia condition, enhanced expression of HIF-1α can be observed with the brightest green fluorescence. However, upon treating with MC-r, the expression of HIF-1α was significantly down-regulated with the increase of MC-r concentration from 25 to 50 ppm (Fig. 5c), suggesting that efficient oxygenation induced by MC-r attenuated the hypoxic condition. Almost no green fluorescence signal was observed when using rabbit IgG as the isotype control, demonstrating the specific binding of HIF-1α antibody (Supplementary Fig. 12). Furthermore, Western blot analysis was employed to study the expression of HIF-1α protein under various conditions. The Western blot data showed a similar tendency as compared with immunofluorescence results (Fig. 5d and Supplementary Fig. 13). All groups exhibited a similar level of tubulin amount, indicating high hypoxia amelioration ability of MC-r without disturbing the cytoskeleton. The intracellular O_2_ generation capacity was also studied using an intracellular O_2_ level indicator [Ru(dpp)_3_]Cl_2_. As shown in Fig. 5e, under hypoxia condition, the group treated with MC-r showed much weaker fluorescence compared to the hypoxia control group, indicating significant intracellular O_2_ supply by MC-r via the single-atom based catalysis. Notably, results also demonstrated a concentration-dependent intracellular O_2_ generation ability of MC-r and hence the hypoxia condition could be easily modulated. These phenomena collectively validated the ability of MC-r to conduct in situ H_2_O_2_ decomposition and intracellular O_2_ generation.

### Enhanced in vitro PDT

**Fig. 6:**
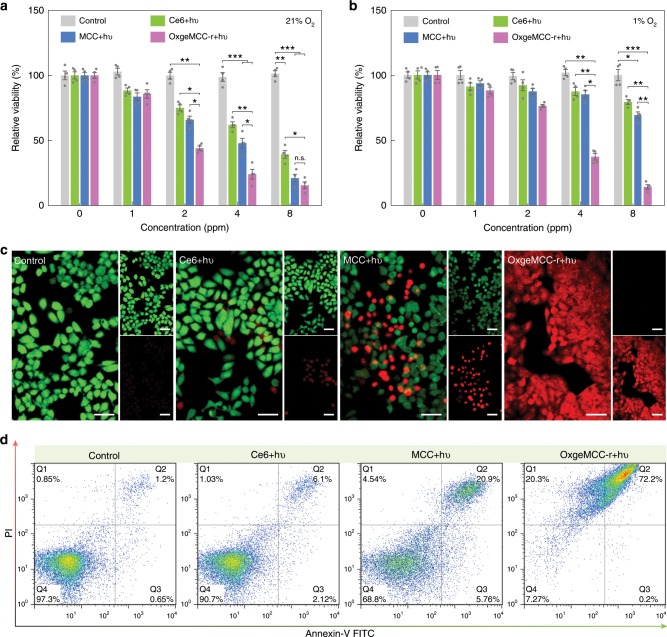
In vitro PDT evaluation of OxgeMCC-r SAE on 4T1 tumor cells. Cell viability assay of free Ce6, MCC, and OxgeMCC-r SAE treated 4T1 cells in **a** normoxic and **b** hypoxic conditions under 671 nm light irradiation (concentration of Ce6: 8 ppm; 671 nm laser power density: 100 mW cm^−2^; irradiation time: 30 s). Data are presented as mean ± s.e.m. (*n* = 4). Statistical analysis was performed via one-way ANOVA. *p < 0.05, **p < 0.01, ***p < 0.001. **c** Live/dead staining of 4T1 cells treated with PBS, free Ce6, MCC, and OxgeMCC-r SAE in the presence of 671 nm laser irradiation under hypoxic conditions. Green signal from calcein AM indicates live cells and red signal from propidium iodide (PI) indicates dead cells (concentration of Ce6: 8 ppm). Scale bar is 50 µm. **d** Cell death mechanism after the treatment of PBS, free Ce6, MCC, and OxgeMCC-r SAE in the presence of 671 nm laser irradiation under hypoxic conditions assessed with Annexin V-FITC/PI by flow cytometry.

Encouraged by the intracellular O_2_ generation, the enhanced PDT efficiency of OxgeMCC-r SAE was assessed against 4T1 cell line. Before which, the subcellular localization of MCC and OxgeMCC-r SAE were studied by confocal laser scanning microscopy. Results indicated that both MCC and OxgeMCC-r SAE localized in endosomes/lysosomes (Supplementary Fig. 14). Furthermore, cell viability results demonstrated that MC-r, MCC, and OxgeMCC-r showed negligible cytotoxicity to 4T1, HeLa, and non-cancerous HEK 293 cell lines under the tested concentrations in the dark condition (Supplementary Figs. 15 and 16). When applied NIR irradiation, both MCC and OxgeMCC-r groups under normoxic condition exhibited comparable phototoxicity toward 4T1 cells at a wide range of Ce6 concentrations using standard 3-(4,5-dimethylthiazol-2-yl)-2,5-diphenyltetrazolium bromide (MTT) method (Fig. 6a). That is to say, the O_2_ supply is sufficient for these tested groups under normoxic condition. It should be noted that higher phototoxicity for MCC and OxgeMCC-r groups as compared with free Ce6 group was due to the enhanced intracellular delivery of Ce6 with the assistance of the self-assembled system. As the hypoxia condition is an intrinsic property of the solid tumor, a mimetic hypoxic condition was achieved through culturing cancer cells within an incubator furnished with hypoxic atmosphere (N_2_/CO_2_/O_2_: 94/5/1 in volume ratio) for 2 h before treatment. Under hypoxic condition, the PDT cytotoxicity of free Ce6 and MCC groups was only 18.4% and 25.3% respectively, due to insufficient O_2_ supply (Fig. 6b). Remarkably, when incubated with OxgeMCC-r (26.7 ppm, containing 8 ppm of Ce6 and 18.7 ppm of MC-r according to the weight ratio) followed by 671 nm laser irradiation, nearly 90% of cancer cells were killed. The augmented PDT efficacy was accounted to sufficient O_2_ supply generated via the single-atom based catalysis with overexpressed intracellular H_2_O_2_^54^. Considering the limited mitochondrial activity under hypoxic condition that may affect the MTT results, another independent CellTiter-Fluor assay that measures the protease activity within live cells was also conducted. As shown in Supplementary Fig. 17, the in vitro PDT results were basically consistent with the MTT data.

The in vitro tumor cell killing efficiency under the mimetic hypoxic condition was further confirmed by calcein-AM (green, live cells) and propidium iodide (red, dead cells) double-staining. With the treatment of free Ce6 or MCC under hypoxia condition, relatively low red fluorescence was observed (Fig. 6c), suggesting inadequate PDT efficiency. In contrast, OxgeMCC-r group presented the highest percentage of dead cells, which was consistent with the results from the MTT assay results. Furthermore, the lethal mechanism was investigated through flow cytometry analysis. Compared to control group, both free Ce6 and MCC groups showed small migration from Q4 (live cells) to Q2 (early apoptosis) and Q3 (late apoptosis), indicating the apoptosis-induced cell death pathway (Fig. 6d). In contrast, the apoptosis percentage of the OxgeMCC-r group presented significantly high value of 72.4% than that of Ce6 group (8.2%) and MCC group (26.8%). In addition, intracellular ROS generation was measured using 2′,7′-dichlorodihydrofluorescein diacetate (H_2_-DCFDA) as a ROS detector. Under hypoxic condition, 4T1 cells treated with OxgeMCC-r SAEs exhibited an enhanced ROS stress level as compared with group treated with MCC nanoparticles (Supplementary Fig. 18). All the above results demonstrated that OxgeMCC-r SAEs could ameliorate hypoxia condition and play an important role in sustaining dependable PDT efficiency.

### MR imaging ability

**Fig. 7:**
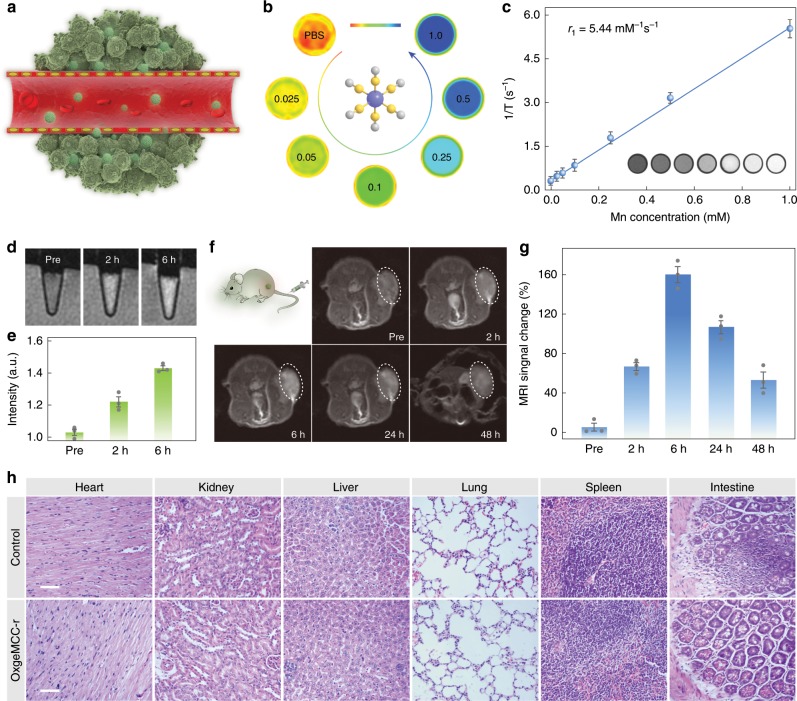
In vitro/vivo MR imaging and biocompatibility of OxgeMCC-r SAE. **a** Accumulation of OxgeMCC-r SAE at the tumor site through the EPR effect. **b** In vitro T_1_-weighted magnetic resonance images of OxgeMCC-r SAE in aqueous solution with various Mn concentrations (mM). **c** Transverse relativity (r_1_) value of 5.44 mM^−1^ s^−1^ for OxgeMCC-r SAE. Inset is magnetic resonance phantom images of OxgeMCC-r SAE. Data are presented as mean ± s.e.m. (*n* = 3). **d** T_1_-weighted MR imaging of 4T1 cells treated with PBS, OxgeMCC-r SAE for 2 h, and OxgeMCC-r SAE for 6 h. **e** Corresponding relative MR imaging intensity of (**d**). Data are presented as mean ± s.e.m. (*n* = 3). **f** In vivo T_1_-weighted magnetic resonance images of 4T1 tumor-bearing mouse at various time points post-injection. Tumor regions are marked with white dashed lines. **g** Quantitative T_1_-weighted MR imaging signals within the tumor site. Data are presented as mean ± s.e.m. (*n* = 3). **h** Micrographs of major organs stained with H&E. Scale bar is 50 µm.

In light of excellent results from above in vitro studies, OxgeMCC-r SAE was applied on animal models in vivo. Due to the fact that Mn is coordinated to six nitrogen atoms forming high-spin Mn–N_6_ (S = 5/2) species, it is no doubt that OxgeMCC-r SAE can be a suitable agent for MR imaging. Thus, we investigated the in vivo MR imaging performance of OxgeMCC-r SAE on a subcutaneous tumor model (Fig. 7a). For diagnostic purpose, we first tested the MR imaging functions of OxgeMCC-r SAE in physiological condition. The T_1_-weighted magnetic resonance images of OxgeMCC-r obtained with a 3.0-T magnetic resonance clinical scanner (GE HDxt) demonstrated a concentration-dependent signal enhancement effect (Fig. 7b, c). The corresponding longitudinal relaxivity (r_1_) value was quantitatively calculated to be 5.44 mM^−1^ s^−1^, which was higher than clinically Gd-based contrast agent (Magnevist, r_1_ = 4.56 mM^−1^ s^−1^)^55^. Thereafter, time-dependent MR imaging ability in 4T1 cells incubated with OxgeMCC-r was assessed. Upon increasing the incubation time, brighter imaging intensity was observed on account of increased cell endocytosis (Fig. 7d). The quantification results demonstrated that OxgeMCC-r SAE after the incubation for 6 h had a significantly higher value than that of 2 h (Fig. 7e). More importantly, in vivo positive signal enhancement within 48 h post-injection suggested that the OxgeMCC-r SAE could be accumulated in the tumor site through the enhanced permeability and retention (EPR) effect^56^. Simultaneously, MR imaging signals were detected in the tumor region, reaching the maximum after 6 h of intravenous injection through the tail vein, suggesting time-dependent tumor accumulation behavior of OxgeMCC-r SAEs (Fig. 7f). Quantitative MR imaging signals within the tumor site treated by OxgeMCC-r SAE further confirmed the findings (Fig. 7g). The long-lasting imaging ability of the OxgeMCC-r SAE would be very useful for guiding in vivo therapy.

### In vivo biocompatibility

The biocompatibility of OxgeMCC-r SAE was further investigated. The hemolysis tests were performed to study the biocompatibility of OxgeMCC-r SAE in blood (Supplementary Fig. 19). Results showed that the hemolysis percentage was less than 2% even at an incubation concentration as high as 1000 ppm. Next, histological examination of various major organs (heart, kidney, liver, lung, spleen, and intestine) from mice injected with OxgeMCC-r SAEs and PBS (as control) was conducted to investigate the potential biological toxicity (Fig. 7h). There was no obvious pathological abnormality or inflammation observed from the mice for both groups. In addition, blood biochemical and blood routine analysis were also performed to understand long-term biosafety of OxgeMCC-r SAE. The blood was collected at different time points after the administration of OxgeMCC-r SAEs. For blood biochemistry, all functional markers, including blood urea nitrogen (BUN), alkaline phosphatase (ALP), alanine transaminase (ALT), and aspartate aminotransferase (AST) were measured. The aminotransferase levels at days 8 and 16 showed no apparent change for the tested groups, indicating compatible kidney and hepatic property of OxgeMCC-r SAEs (Supplementary Table 2). For blood routine analysis, results revealed the tested nine common indexes are within the normal ranges, indicating negligible blood toxicity of OxgeMCC-r SAE under the treatment dose within 16 days in vivo (Supplementary Table 3)^57^. Overall, these results firmly demonstrated the in vivo biocompatibility of OxgeMCC-r SAE, potentiating its further application as a theranostic agent.

### In vivo PDT

**Fig. 8:**
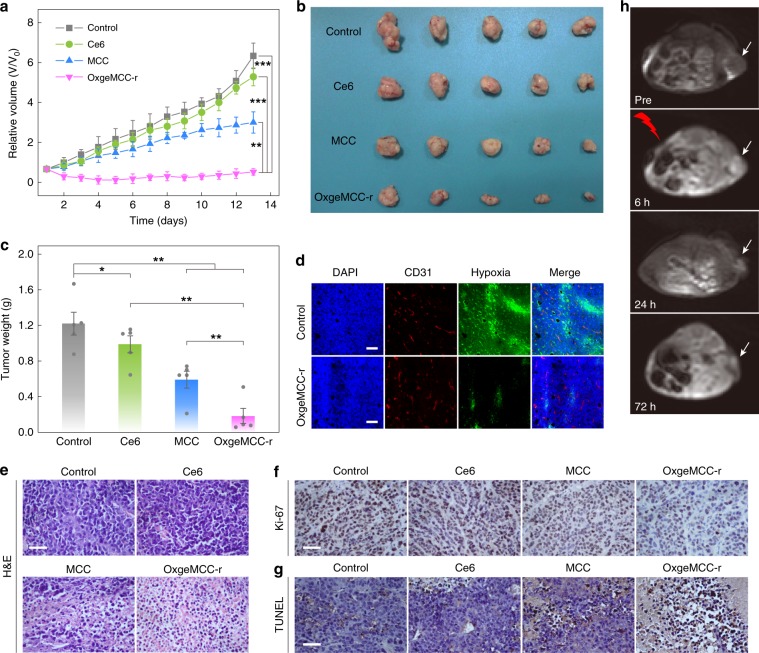
In vivo PDT efficiency assessments. **a** Relative tumor volumes of mice after various treatments (control, Ce6, MCC, and OxgeMCC-r, *n* = 5). The last three groups were treated with laser irradiation: 5 min with a power density of 100 mW cm^−2^. Injection dose is 100 µL, with a Ce6 concentration of 4 mg kg^−1^. **b** Photographic images of tumors excised from different groups after various treatments indicated. **c** Average weights of tumors from different groups of mice after various treatments indicated. **d** Representative hypoxia immunofluorescence images of tumor slices. Nuclei, blood vessels, and hypoxic regions were stained by 4’,6-diamidino-2-phenylindole (DAPI, blue), anti-CD31 antibody (red), and anti-pimonidazole antibody (green), respectively. Scale bar is 100 µm. **e** H&E staining and **f** Ki-67 staining of tumor slices from different groups after various treatments. Scale bar is 50 µm. **g** TUNEL staining of tumor slices from different groups indicated. Scale bar is 50 µm. **h** MR imaging of tumor-bearing mouse at different treatment points. Data are presented as mean ± s.e.m. (*n* = 5). Statistical analysis was performed via one-way ANOVA. *p < 0.05, **p < 0.01, ***p < 0.001.

Encouraged by the superior in vitro PDT efficacy, satisfactory biocompatibility as well as high tumor accumulation, in vivo PDT of OxgeMCC-r SAE was carried out on 4T1 tumor-bearing mice. The mice were randomly divided into four groups (control, free Ce6, MCC, and OxgeMCC-r, *n* = 5). For mice treated with light irradiation, the animals were subjected to irradiation (671 nm laser, 100 mW cm^−2^, 5 min) at 6 h post intravenous injection. Our results clearly showed the control group exhibited the fastest tumor growth (Fig. 8a). Similar results were also observed for free Ce6 group with no noticeable tumor growth inhibition under laser irradiation due to fast metabolism of the free drug form. The tumor growth in mice administrated with MCC could be partially inhibited on account of the efficient delivery of Ce6 to tumor sites (Fig. 8b). Excitingly, the group treated with OxgeMCC-r SAEs showed remarkable tumor suppression under same dosage of laser irradiation. During the treatments, mice from all groups showed the same increasing trend in body weights and no obvious changes in the food and water intake, indicating superior safety of MC and MC-r for delivering theranostic agents in vivo (Supplementary Fig. 20). At the end of treatment (day 14), mice from all groups were euthanized and tumor tissues were harvested. Results revealed the average weight of tumor tissues obtained by anatomy for OxgeMCC-r group was the lowest, at only 0.19 g (Fig. 8c). In comparison to the MCC group, the therapeutic efficacy achieved by OxgeMCC-r SAE group was exceptional, demonstrating a synergistic effect owing to the increase in the localized availability of O_2_ for self-sustained photodynamic therapy.

The immunofluorescence staining was performed to further confirm the capability of OxgeMCC-r SAE for in situ amelioration of hypoxia status within tumor. For doing this, pimonidazole hydrochloride was first employed as a hypoxia probe to test the hypoxia status of tumor tissues^52^. Tumor hypoxia and blood vessels were then stained with anti-pimonidazole antibody (green signals) and anti-CD31 antibody (red signals), respectively. Results suggested group treated with OxgeMCC-r SAEs displayed significantly reduced hypoxia signals. In contrast, the control group exhibited bright green hypoxia signals (Fig. 8d). It could be concluded that tumor hypoxia condition was alleviated due to the catalysis of H_2_O_2_ into O_2_ in the presence of OxgeMCC-r SAEs. Next, we performed hematoxylin and eosin (H&E) staining of tumor slices from different groups to assess the therapeutic efficiency (Fig. 8e). Among all the conducted groups, mice treated with OxgeMCC-r SAEs under laser irradiation suffered the most prominent tumor tissue damage as noticed, validating its high PDT effect. Furthermore, Ki-67 and TUNEL staining were conducted on tumor slices to study the tumor cell proliferation and apoptosis levels, respectively. The OxgeMCC-r SAE group exhibited the least brown area, indicating its excellent inhibiting effect on the proliferation of tumor cells (Fig. 8f). As expected, the highest level of positive TUNEL staining signals on tumor slices was detected from the OxgeMCC-r SAE group, which was consistent with the H&E and Ki-67 staining results (Fig. 8g)^58^.

### In vivo MR imaging-guided PDT

As a proof of concept for being a theranostic nanoplatform, we performed MR imaging for real-time observation of the tumor growth on 4T1 tumor-bearing mouse after administration of OxgeMCC-r SAEs followed by PDT. After the accumulation in the tumor sites, OxgeMCC-r could effectively integrate tumor PDT and prolonged MR imaging in a spatiotemporal manner. As shown in Fig. 8h, magnetic resonance images showed significant OxgeMCC-r accumulation within the tumor site (indicated by the white arrow) at 6 h post injection. After which, the tumor sites received 671 nm laser irradiation. Noticeable shrinkage of tumor volume was observed 24 h after injection (18 h after PDT). Furthermore, almost complete tumor elimination at 3 days after the PDT treatment was observed through MR imaging results. Thus, the developed OxgeMCC-r SAE could be a potential theranostic nanoplatform for MR imaging guided tumor PDT.

## Discussion

Catalysis-based nanomedicine has received much research attention, since this approach shows superior therapeutic efficacy in the cancer treatment. By using MOFs as single-atom anchoring materials, we have successfully developed a multifunctional OxgeMCC-r single-atom nanozyme through one-step self-assembly strategy. OxgeMCC-r SAE with 2.23 wt% single-atom Ru loading content was capable of degrading intracellular H_2_O_2_ to O_2_ to relieve the hypoxia condition of solid tumor, leading to an enhanced ROS generation, and finally causing apoptotic cell death both in vitro and in vivo. Unlike traditional drug carriers, OxgeMCC-r SAE presents a high loading capacity of Ce6 photosensitizer. OxgeMCC-r SAE could selectively accumulate within tumor sites for enhanced PDT of cancer under the guide of T_1_ MR imaging. Having high loading capacity for Ce6, superior catalytic property, and suitable imaging capability for in vivo tracking, the OxgeMCC-r SAE would be a promising anticancer theranostic agent, advancing further development of different single-atom nanozymes in the field of catalytic nanomedicine.

## Methods

### Preparation of OxgeMCC-r SAE

For the synthesis of OxgeMCC-r SAEs, K_3_[Co(CN)_6_] powder (166 mg) was dispersed in ultrapure water (20 mL) with a final concentration of 8.3 mg/mL. RuCl_3_ solution (10 mM) was also prepared using ultrapure water. The hydrophobic drug Ce6 (60 mg) was dissolved in ethanol (15 mL). Then, Mn(CH_3_COO)_2_·4H_2_O (25 mM, 30 mL) aqueous solution, RuCl_3_ aqueous solution (5 mL) and Ce6 (15 mL) ethanol solution were mixed to form a homogeneous solution. Using PVP (5 mg/mL) as the surfactant, K_3_[Co(CN)_6_] solution (20 mL) was dropwise added to the mixed solution using a syringe with the total volume reaching about 70 mL under magnetic stirring. Turbidity generated immediately upon the addition of K_3_[Co(CN)_6_] solution. The final concentration of Mn(II), Ce6, Ru(III), K_3_[Co(CN)_6_], and PVP was 0.59, 0.86, 0.072, 2.37, and 5 mg mL^−1^, respectively. By mixing the above solution with NaBH_4_ (10 mL, 1 mg/mL) solution and stirring for another 12 h, the OxgeMCC-r SAE was obtained. The fabrication of MC-r nanoparticles was achieved without the addition of Ce6. The synthesis of MCC nanoparticles was achieved without the addition of Ru(III), and for MC nanoparticles without the addition of both Ce6 and Ru (III). To adjust the drug loading capacity, the concentration of the initial Ce6 was varied from 0 to 120 mg. The final products were centrifuged at 16099 × *g* for 30 min, washed thrice with water and dispersed for further characterizations.

### Decomposition of H_2_O_2_

The catalytic effect of MC-r and OxgeMCC-r toward hydrogen peroxide was tested by mixing MC-r (2 mM) or OxgeMCC-r (2 mM) with H_2_O_2_ (1 mM) in PBS at room temperature. At predetermined time points, the solution (50 µL) was collected and added to Ti(SO_4_)_2_ solution (100 µL)^21^. The content of H_2_O_2_ was calculated through measuring the UV-vis absorbance at 405 nm. For verifying the catalytic durability of OxgeMCC-r SAE toward H_2_O_2_, H_2_O_2_ solution was added repeatedly to the OxgeMCC-r SAE solution followed by measuring the catalytic efficiency under pH value of 6.5. An optical oxygen sensor (NeoFox, Ocean Optics, Inc.) was used to quantify the amount of evolving oxygen in the reaction system (150 µM of H_2_O_2_ and 250 µM of OxgeMCC-r). For comparison, MnO_2_ nanoparticles were also synthesized. Typically, bovine serum albumin (100 mg) was added into MnCl_2_ solution (10 mL, 10 mM). Thereafter, sodium hydroxide solution (1.0 M) was added dropwise into the mixed solution to adjust the pH value to 11. After vigorous stirring for 4 h, MnO_2_ nanoparticles were obtained. The catalytic durability of MnO_2_ nanoparticles toward H_2_O_2_ was also studied under pH value of 6.5.

### Singlet oxygen detection

A singlet oxygen indicator 1,3-diphenylisobenzonfuran (DPBF, Sigma-Aldrich) was used to study ^1^O_2_ generation by measuring the quenching UV-vis absorption at 421 nm. The test solution was prepared by adding DPBF (30 μL) in DMSO (10 mM) to OxgeMCC-r solution (3 mL, 10 µg mL^−1^). Before the irradiation (671 nm, 100 mW cm^−2^, 30 s, light dose is 0.1 J cm^−2^ s^−1^), the mixed solution was saturated with argon atmosphere under dark environment to achieve hypoxic condition. Upon the irradiation, the absorption intensity of DPBF was recorded every 2 min. As controls, DPBF absorption was recorded in PBS, and MCC solution plus H_2_O_2_ with or without 671 nm irradiation.

### In vitro PDT

Three cell lines including human embryonic kidney normal cells (HEK 293), human cervical cancer cells (HeLa), and murine breast cancer cells (4T1) were originally obtained from American Type Culture Collection (ATCC). Three cell lines were seeded in 96-well plates with an initial seeding density of 1 × 10^4^ cells per well and incubated in 5% CO_2_ at 37 °C for 24 h. For the biocompatibility study, the cells were incubated with MC-r, MCC, and OxgeMCC-r SAEs at different concentrations in the dark condition for 24 h. For in vitro therapy, 4T1 cancer cells were incubated with free Ce6, MCC, and OxgeMCC-r at a range of concentrations at 37 °C under normoxic or hypoxic condition for 24 h. The hypoxic condition was achieved by culturing cells in an incubator supplied with hypoxic atmosphere (N_2_/CO_2_/O_2_: 94/5/1 in volume ratio). After 4 h incubation, the cells were irradiated with a 671 nm laser (100 mW cm^−2^, 30 s). The relative cell viability was measured using the standard MTT assay. Typically, the sample-containing medium was replaced with MTT solution (5 μg mL^−1^, 10 μL) and incubated for 4 h. Finally, DMSO (100 μL) was added into each well to dissolve the purple formazan crystals before measuring the UV-vis absorbance. Another mitochondrial activity-independent CellTiter^TM^ Fluor cell viability assay (Promega Pte Ltd.) was also performed to study the in vitro therapeutic efficiency under both normoxic and hypoxic conditions. The dark toxicity was evaluated similarly but without light irradiation.

### Live/dead cell staining assay

4T1 cancer cells were incubated in 6-well plates under hypoxic condition at 37 °C for 24 h. After which, previous medium was replaced by fresh medium that contained free Ce6, MCC, and OxgeMCC-r (Ce6 concentration is 8 ppm) for 4 h co-culture. Thereafter, the cells were treated with a 671 nm laser irradiation (100 mW cm^−2^, 30 s). After co-culture for another 4 h, cells were stained with calcein AM (5 μL) and PI (10 μL) according to the product description for 0.5 h and then observed using a fluorescence inverted microscope (Olympus UHGLGPS, China).

### Apoptosis and necrosis assay

4T1 cancer cells were seeded in 6-well plates under hypoxic condition at 37 °C for 24 h. Subsequently, previous medium was replaced by fresh one with free Ce6, MCC, and OxgeMCC-r ([Ce6] is 8 ppm). After 4 h, the cells were irradiated with laser irradiation (671 nm, 100 mW cm^−2^, 30 s). After co-culture for another 4 h, all treated cells were harvested and quantified by apoptosis with an annexin V-FITC/PI apoptosis detection kit using flow cytometer (Guava EasyCyte 6-2L).

### Intracellular ROS detection

4T1 cells were seeded with a density of 3.6 × 10^4^ per well in a confocal dish under hypoxic condition. After the incubation at 37 °C for 24 h, previous medium was replaced by fresh one containing PBS, MCC, or OxgeMCC-r ([Ce6] is 8 ppm) in the dark, followed by the addition of cell-permeant 2’,7’-dichlorodihydrofluorescein diacetate (H_2_-DCFDA, 25 µM). After 4 h, the cells were treated with laser irradiation (671 nm, 100 mW cm^−2^, 30 s). After culturing for another 4 h, all treated groups were then observed using laser scanning confocal microscope (ZEISS, LSM710).

### Intracellular hypoxia immunofluorescence

4T1 cells were seeded in confocal dish. Following different treatments, cells were fixed with 1% paraformaldehyde at 37 °C for 10 min and permeabilized with PBS containing 0.2% Triton X-100 at 37 °C for 1 min. Subsequently, the blocking step was carried out with PBST (PBS with 0.05% Tween-20) buffer containing 1% bovine serum albumin at room temperature for 45 min. Finally, the cells were incubated with anti-HIF-1α (Cat. no. 36169T, Cell Signaling Technology) and anti-α-Tubulin (Cat. no. T9026, Sigma-Aldrich) primary antibodies in a humidified chamber at 4 °C overnight, followed by corresponding fluorescence labeled secondary antibodies at room temperature for 1 h. Images were acquired with a DeltaVision softWoRx software (Applied Precision) and processed by deconvolution and z-stack projection.

### Western blot

4T1 cells were first treated with MC-r before incubation under normoxic or hypoxic condition. After which, cells were lysed and collected, followed by mixing with sample buffer and heated at 95 °C for 5 min. After the purification with 10 % sodium dodecyl sulfate-polyacrylamide gel electrophoresis, the collected protein was transferred to a polyvinylidene difluoride membrane. The sample was stained with primary antibodies anti-HIF-1α (Cat no. 36169T, Cell Signaling Technology) for evaluating the degree of hypoxia and anti-α-Tubulin (Cat. no. T9026, Sigma-Aldrich) as the loading control, and then with horseradish peroxidase-labeled secondary antibodies. The HIF-1α level was monitored by enhanced chemiluminescence using Gel Doc system (Bio-Rad).

### Intracellular O_2_ generation

4T1 cells were first seeded on cover slides and incubated with PBS or MC-r under normal or hypoxic condition for 12 h. Following which, cells were treated with an oxygen indicator [Ru(dpp)_3_]Cl_2_ (10 μg/mL, ThermoFisher) and further incubated for 12 h. The samples were removed and cells were washed thrice with PBS to remove any residual MC-r nanoparticles and free [Ru(dpp)_3_]Cl_2_ molecules. Fluorescence images of [Ru(dpp)_3_]^2+^ (*λ*_ex_ = 450 nm, *λ*_em_ = 610 nm) relating to the intracellular O_2_ level was obtained using a confocal laser scanning microscope.

### MR imaging

OxgeMCC-r SAE solutions with different Mn concentrations were measured with a clinical MR imaging scanner (GE HDxt, 3.0-T) from the First Affiliated Hospital of Anhui Medical University. For in vitro MR imaging, 4T1 cells were pretreated with 18.4 ppm OxgeMCC-r SAE. After incubation for 2 and 6 h, 5 × 10^6^ cells were washed with PBS three time and collected for MR imaging test. For in vivo MR imaging, 4T1 tumor-bearing mice (*n* = 3) weighing 20 g on average (SLAC Laboratory animal Co., Ltd., Shanghai) were employed for the study. Animal experiments were conducted under the animal guidelines authorized by the Animal Care Committee (University of Science and Technology of China) and complied with all relevant ethical regulations. T_1_-weighted magnetic resonance images were acquired pre-injection as well as 2 h, 6 h, 24 h, and 48 h post-injection of OxgeMCC-r saline solution (13.2 mg kg^−1^). Fast spin echo multi-slice (f-SEMS) sequence was used for the acquisition of the T_1_-weighted magnetic resonance images.

### In vivo biocompatibility analysis

For the long-term biocompatibility study, mice were first intravenously injected with OxgeMCC-r SAE. Two-week later, the mice were euthanized and their main organs (heart, kidney, liver, lung, spleen, and intestine) were collected and fixed with 4% paraformaldehyde. After embedded in paraffin, tissue samples were cryo-sliced (4 μm) before further histological analysis by standard hematoxylin and eosin (H&E) staining procedure.

### In vivo PDT

4T1 tumor-bearing mice were randomly allocated into four groups (*n* = 5): control, free Ce6, MCC, and OxgeMCC-r SAE when the average tumor volume reached 100 mm^3^. Ce6, MCC and OxgeMCC-r in saline solutions (injection dose = 100 µL, at a Ce6 concentration of 4 mg kg^−1^) were injected via the tail vein. For comparison, saline (100 µL) was injected into mice as the control group. At 6 h post injection, the tumors from groups 2-4 were irradiated with a 671 nm laser (100 mW cm^−2^, 5 min). Tumor sizes were measured every day since the start of treatment, and the tumor volume was calculated according to the equation: Volume = (Tumor length) × (Tumor width)^2^/2 (mm^3^). Relative tumor volume was normalized to its initial size before the treatment. At the end of the treatment, all mice were sacrificed, and tumors were collected and weighed.

### Immunofluorescence staining

The variation of hypoxia degree within tumor microenvironment after 24 h post injection of OxgeMCC-r SAE was investigated with immunofluorescence staining. At 90 min before tumors were surgically excised, pimonidazole hydrochloride (Hypoxyprobe-1 plus kit, Hypoxyprobe, USA) was intraperitoneally injected into mice at a dose of 30 mg kg^−1^. The collected tumor slices were firstly stained with mouse anti-pimonidazole monoclonal antibody (dilution 1:200, Hypoxyprobe) and rat-anti mouse CD31 primary antibody (dilution 1:200, Biolegend) to mark tumor hypoxia regions and blood vessels, respectively. Thereafter, the slices were stained with Alex 488-conjugated goat anti-mouse secondary antibody (Jackson Inc.) and rhodamine-conjugated donkey anti-rat secondary antibody (Jackson Inc.), respectively. The nuclei were stained with 4’,6-diamidino-2-phenylindole (DAPI, Invitrogen). The final images of stained slices were obtained using a confocal laser scanning microscope (Zeiss LSM 710).

### Pathological investigation

After in vivo therapy, tumor tissues of control and OxgeMCC-r groups were resected, fixed with 4% formaldehyde solution and embedded in paraffin blocks. The tissue blocks were sectioned and stained with hematoxylin and eosin (H&E). For Ki-67 staining, tissue sections were stained with anti-Ki-67 polyclonal antibody (ab15580, Abcam, USA) at 4 °C overnight before imaging. Terminal deoxynucleotidyl transferase (TdT)-mediated deoxyuridine triphosphate (dUTP) nick end labeling (TUNEL) staining was conducted using in situ cell death detection kit (POD, Roche, America). All processes were carried out following the standard protocol.

### Statistical analysis

All data were expressed as mean ± standard error of the mean. The statistical difference between different groups of data was evaluated by one-way ANOVA, and *p* < 0.05 was considered to be statistically significant. Asterisk (*) denotes statistical significance between bars (*p < 0.05, **p < 0.01, ***p < 0.001) conducted using GraphPad Prism 6.0.

### Reporting summary

Further information on research design is available in the Nature Research Reporting Summary linked to this article.

## Supplementary information

Supplementary Information

Peer Review

Reporting Summary

## Data Availability

The authors declare that the data supporting the findings of this study are available within the article and its Supplementary Information. Extra data are available from the corresponding author upon reasonable request.

## References

[1] Felsher DW (2003). Cancer revoked: oncogenes as therapeutic targets. Nat. Rev. Cancer.

[2] Lucky SS, Soo KC, Zhang Y (2015). Nanoparticles in photodynamic therapy. Chem. Rev..

[3] Hopper C (2000). Photodynamic therapy: a clinical reality in the treatment of cancer. Lancet Oncol..

[4] Lovell JF, Liu TWB, Chen J, Zheng G (2010). Activatable photosensitizers for imaging and therapy. Chem. Rev..

[5] Tian B, Wang C, Zhang S, Feng L, Liu Z (2011). Photothermally enhanced photodynamic therapy delivered by nano-graphene oxide. ACS Nano.

[6] Abbas M, Zou Q, Li S, Yan X (2017). Self-assembled peptide- and protein-based nanomaterials for antitumor photodynamic and photothermal therapy. Adv. Mater..

[7] Xu S (2018). Oxygen and Pt(II) self-generating conjugate for synergistic photo-chemo therapy of hypoxic tumor. Nat. Commun..

[8] Zhang C (2015). Marriage of scintillator and semiconductor for synchronous radiotherapy and deep photodynamic therapy with diminished oxygen dependence. Angew. Chem. Int. Ed..

[9] Xu R (2016). Nanoscale metal-organic frameworks for ratiometric oxygen sensing in live cells. J. Am. Chem. Soc..

[10] Vander Heiden MG, Cantley LC, Thompson CB (2009). Understanding the Warburg effect: the metabolic requirements of cell proliferation. Science.

[11] Bristow RG, Hill RP (2008). Hypoxia, DNA repair and genetic instability. Nat. Rev. Cancer.

[12] Yu Z, Zhou P, Pan W, Li N, Tang B (2018). A biomimetic nanoreactor for synergistic chemiexcited photodynamic therapy and starvation therapy against tumor metastasis. Nat. Commun..

[13] Kuang Y, Balakrishnan K, Gandhi V, Peng X (2011). Hydrogen peroxide inducible DNA cross-linking agents: targeted anticancer prodrugs. J. Am. Chem. Soc..

[14] Szatrowski TP, Nathan CF (1991). Production of large amounts of hydrogen peroxide by human tumor cells. Cancer Res..

[15] Lambeth JD (2004). NOX enzymes and the biology of reactive oxygen. Nat. Rev. Immunol..

[16] Fan W (2015). Intelligent MnO_2_ nanosheets anchored with upconversion nanoprobes for concurrent pH-/H_2_O_2_-responsive UCL imaging and oxygen-elevated synergetic therapy. Adv. Mater..

[17] Huang CC (2016). An implantable depot that can generate oxygen in situ for overcoming hypoxia-induced resistance to anticancer drugs in chemotherapy. J. Am. Chem. Soc..

[18] Jia Q (2018). A magnetofluorescent carbon dot assembly as an acidic H_2_O_2_-driven oxygenerator to regulate tumor hypoxia for simultaneous bimodal imaging and enhanced photodynamic therapy. Adv. Mater..

[19] Wang H (2018). Photosensitizer-crosslinked in-situ polymerization on catalase for tumor hypoxia modulation & enhanced photodynamic therapy. Biomaterials.

[20] Kim J (2017). Continuous O_2_-evolving MnFe_2_O_4_ nanoparticle-anchored mesoporous silica nanoparticles for efficient photodynamic therapy in hypoxic cancer. J. Am. Chem. Soc..

[21] Wang D (2018). Photo-enhanced singlet oxygen generation of Prussian blue-based nanocatalyst for augmented photodynamic therapy. iScience.

[22] Yang G (2017). Hollow MnO_2_ as a tumor-microenvironment-responsive biodegradable nano-platform for combination therapy favoring antitumor immune responses. Nat. Commun..

[23] Wei S (2018). Direct observation of noble metal nanoparticles transforming to thermally stable single atoms. Nat. Nanotechnol..

[24] Wang A, Li J, Zhang T (2018). Heterogeneous single-atom catalysis. Nat. Rev. Chem..

[25] Lin L (2017). Low-temperature hydrogen production from water and methanol using Pt/alpha-MoC catalysts. Nature.

[26] Liu G (2017). MoS_2_ monolayer catalyst doped with isolated Co atoms for the hydrodeoxygenation reaction. Nat. Chem..

[27] Geng Z (2018). Achieving a record-high yield rate of 120.9 µg_NH3_ mg_cat._^−1^ h^−1^ for N_2_ electrochemical reduction over Ru single-atom catalysts. Adv. Mater..

[28] Gong N (2019). Carbon-dot-supported atomically dispersed gold as a mitochondrial oxidative stress amplifier for cancer treatment. Nat. Nanotechnol..

[29] Xu B (2019). A single-atom nanozyme for wound disinfection applications. Angew. Chem. Int. Ed..

[30] Wang D (2016). Controllable synthesis of dual-MOFs nanostructures for pH-responsive artemisinin delivery, magnetic resonance and optical dual-model imaging-guided chemo/photothermal combinational cancer therapy. Biomaterials.

[31] Cheng G (2018). Self-assembly of extracellular vesicle-like metal-organic framework nanoparticles for protection and intracellular delivery of biofunctional proteins. J. Am. Chem. Soc..

[32] Zheng H (2016). One-pot synthesis of metal-organic frameworks with encapsulated target molecules and their applications for controlled drug delivery. J. Am. Chem. Soc..

[33] Rogge SMJ (2017). Metal-organic and covalent organic frameworks as single-site catalysts. Chem. Soc. Rev..

[34] Wu MX, Yang YW (2017). Metal-organic framework (MOF)-based drug/cargo delivery and cancer therapy. Adv. Mater..

[35] Otake KI (2018). Single-atom-based vanadium oxide catalysts supported on metal-organic frameworks: selective alcohol oxidation and structure-activity relationship. J. Am. Chem. Soc..

[36] Wang D (2017). Core–shell metal-organic frameworks as Fe^2+^ suppliers for Fe^2+^-mediated cancer therapy under multimodality imaging. Chem. Mater..

[37] Wang Y (2015). Universal strategy for homogeneously doping noble metals into cyano-bridged coordination polymers. ACS Appl. Mater. Interfaces.

[38] Jiang P (2018). Tuning the activity of carbon for electrocatalytic hydrogen evolution via an iridium-cobalt alloy core encapsulated in nitrogen-doped carbon cages. Adv. Mater..

[39] Su J (2017). Ruthenium-cobalt nanoalloys encapsulated in nitrogen-doped graphene as active electrocatalysts for producing hydrogen in alkaline media. Nat. Commun..

[40] Cao GJ, Jiang X, Zhang H, Croley TR, Yin JJ (2017). Mimicking horseradish peroxidase and oxidase using ruthenium nanomaterials. RSC Adv..

[41] Zhang Y (2018). Nanozyme decorated metal-organic frameworks for enhanced photodynamic therapy. ACS Nano.

[42] Jv Y, Li B, Cao R (2010). Positively-charged gold nanoparticles as peroxidase mimic and their application in hydrogen peroxide and glucose detection. Chem. Commun..

[43] Wang D (2018). In situ one-pot synthesis of MOF-polydopamine hybrid nanogels with enhanced photothermal effect for targeted cancer therapy. Adv. Sci..

[44] Wang D (2015). Novel Mn_3_[Co(CN)_6_]_2_@SiO_2_@Ag core-shell nanocube: enhanced two-photon fluorescence and magnetic resonance dual-modal imaging-guided photothermal and chemo-therapy. Small.

[45] Tao H (2019). Nitrogen fixation by Ru single-atom electrocatalytic reduction. Chem.

[46] Yabuta H (2014). X-ray absorption near edge structure spectroscopic study of Hayabusa category 3 carbonaceous particles. Earth, Planets Space.

[47] Wang X (2017). Uncoordinated amine groups of metal-organic frameworks to anchor single Ru sites as chemoselective catalysts toward the hydrogenation of quinoline. J. Am. Chem. Soc..

[48] Nowicki A, Le Boulaire V, Roucoux A (2007). Nanoheterogeneous catalytic hydrogenation of arenes: evaluation of the surfactant-stabilized aqueous ruthenium(0) colloidal suspension. Adv. Synth. Catal..

[49] Bonet F (1999). Synthesis of monodisperse Au, Pt, Pd, Ru and Ir nanoparticles in ethylene glycol. Nanostruct. Mater..

[50] Zhang C (2017). Magnesium silicide nanoparticles as a deoxygenation agent for cancer starvation therapy. Nat. Nanotechnol..

[51] Lopes CMA, Felisberti MI (2003). Mechanical behaviour and biocompatibility of poly(1-vinyl-2-pyrrolidinone)–gelatin IPN hydrogels. Biomaterials.

[52] Chen Q (2016). Intelligent albumin-MnO_2_ nanoparticles as pH-/H_2_O_2_-responsive dissociable nanocarriers to modulate tumor hypoxia for effective combination therapy. Adv. Mater..

[53] Salceda S, Caro J (1997). Hypoxia-inducible factor 1α (HIF-1α) protein is rapidly degraded by the ubiquitin-proteasome system under normoxic conditions: its stabilization by hypoxia depends on redox-induced changes. J. Biol. Chem..

[54] Huo M, Wang L, Chen Y, Shi J (2017). Tumor-selective catalytic nanomedicine by nanocatalyst delivery. Nat. Commun..

[55] Hifumi H, Yamaoka S, Tanimoto A, Citterio D, Suzuki K (2006). Gadolinium-based hybrid nanoparticles as a positive MR contrast agent. J. Am. Chem. Soc..

[56] Fang J, Nakamura H, Maeda H (2011). The EPR effect: unique features of tumor blood vessels for drug delivery, factors involved, and limitations and augmentation of the effect. Adv. Drug Deliv. Rev..

[57] Zhu P, Chen Y, Shi J (2018). Nanoenzyme-augmented cancer sonodynamic therapy by catalytic tumor oxygenation. ACS Nano.

[58] Wang D (2016). Magnetically guided delivery of DHA and Fe ions for enhanced cancer therapy based on pH-responsive degradation of DHA-loaded Fe_3_O_4_@C@MIL-100(Fe) nanoparticles. Biomaterials.

