# Supplementary material for: Self-assembled single-atom nanozyme for enhanced photodynamic therapy treatment of tumor
Source: Nat Commun. 2020 Jan 17;11:357. doi: 10.1038/s41467-019-14199-7 (PMC6969186; doi:10.1038/s41467-019-14199-7)
Supplement: Supplementary file 1 — Supplementary Information [file 41467_2019_14199_MOESM1_ESM.pdf]

Supplementary Information

Self-assembled single-atom nanozyme for enhanced photodynamic therapy treatment of tumor

Wang et al.

Supplementary Methods

Materials. Dopamine and potassium cobaltocyanide ($K_3[Co(CN)_6]$) were obtained from Alfa Aesar. Manganese acetate ($Mn(CH_3COO)_2\cdot 4H_2O$), poly(vinylpyrrolidone) (PVP, M_w = 40, 000), and ruthenium(III) chloride trihydrate ($RuCl_3\cdot 3H_2O$) were obtained from Sigma-Aldrich. Chlorin e6 (Ce6) was purchased from Frontier Scientific, Inc. (Salt Lake City, UT, USA). Other chemicals were obtained from Sinopharm Group Co. Ltd. All chemicals were used as received without further purifications.

Characterizations. Transmission electron microscopy (TEM) images were acquired with a transmission electron microscope (Hitachi H-7650, Japan) operated on an accelerating voltage of 100 kV. The high-angle annular dark-field scanning transmission electron microscopy (HAADF-STEM) and corresponding energy-dispersive spectroscopy (EDS) mapping analyses were executed on a Talos F200X STEM. Powder X-ray diffraction (XRD) patterns were acquired on a Rigaku D/MAX-cAX-ray (Japan) diffractometer equipped with Cu Kα radiation. Scanning electron microscopy (SEM) images were measured on a JEOL JSM-6700M (Japan) microscopy. The value of metal content was measured by inductively coupled plasma-atomic emission spectrometer mass spectrometer (ICP-MS, Optima 7300DV, America). Nitrogen adsorption-desorption isotherms at 77 K were measured by surface area analyzer Micromeritics (ASAP 2020) for calculations of BET surface area and DFT pore size distribution. Pore volume was calculated from the adsorbed amounts at P/P_o = 0.985. Ultraviolet-visible (UV-Vis) absorption spectra were measured on a SOLID-3700 (Japan) spectrometer. X-Ray photoelectron spectroscopy (XPS) analysis was performed on ESCALAB 250 (UK) multi-technique X-ray photoelectron spectrometer. The near-edge X-ray absorption fine structure (NEXAFS) of C K-edge and N K-edge was measured at the soft X-ray magnetic circular dichroism end station (XMCD) of National Synchrotron Radiation Laboratory (NSRL) in University of Science and Technology of China. For measurements of ζ-potential and size distribution, a DLS instrument (Nano ZS ZEN3600, Malvern) was used. A 671 nm laser (LSR671ML, Yuanming Ningbo LTD, China) was used for irradiation.

Supplementary Table 1. Component contents.

Ingredient	Initial concentration (mg mL^{-1})	Assembly concentration (mg mL^{-1})	Loading percentage (%)
$[Co(CN)_6]^{3-}$	1.54	0.95	50.47%
Mn^{2+}	0.59	0.32	17.0%
Ru^{3+}	0.072	0.042	2.23%
Ce6	0.86	0.57	30.3%

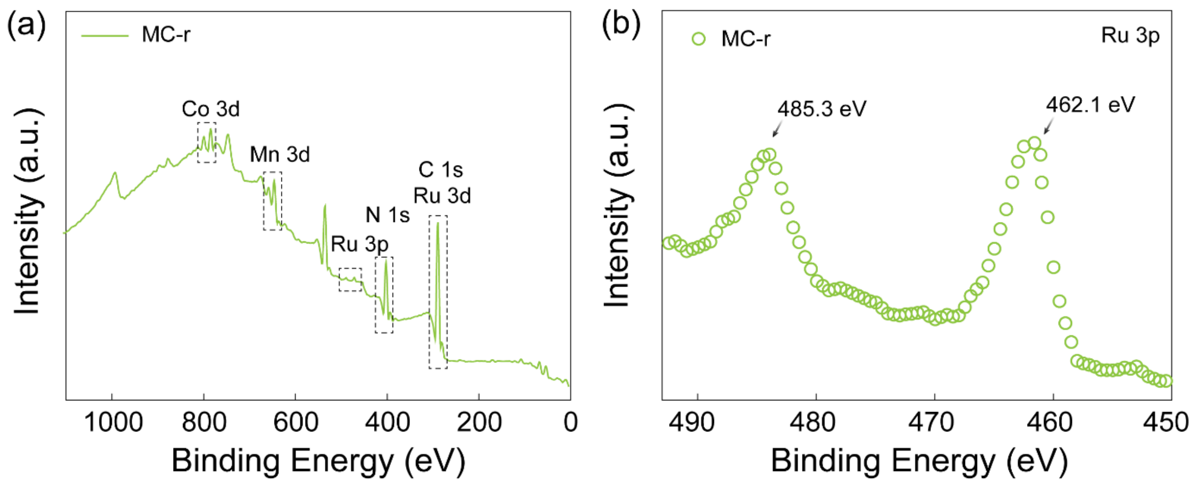

Supplementary Figure 1. XPS characterization of MC-r. (a) XPS spectrum of MC-r. (b) XPS spectra of Ru 3p from MC-r enlarged in Supplementary Figure 1a.

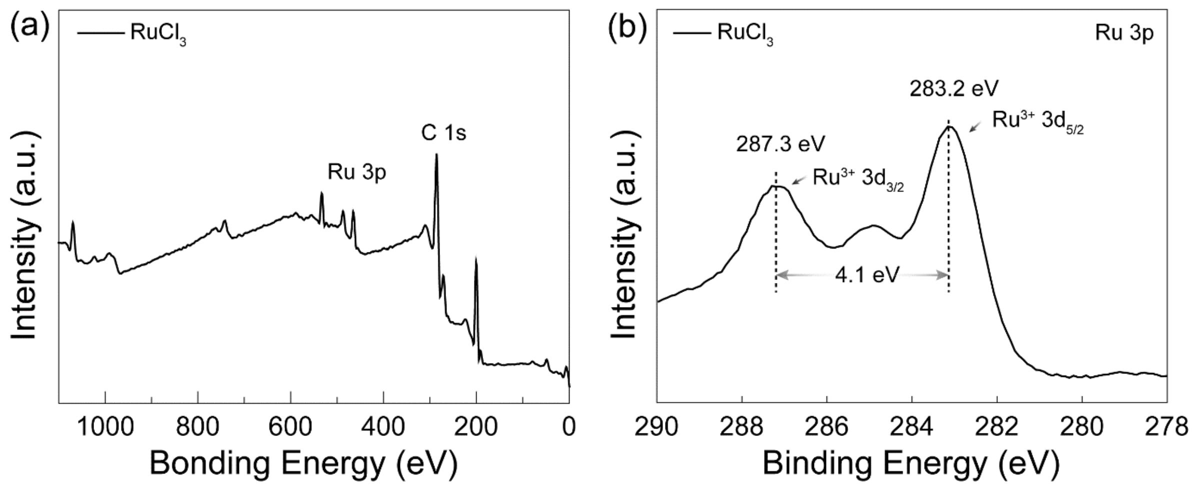

Supplementary Figure 2. XPS characterization of $RuCl_3$ powder. (a) XPS spectrum of pure $RuCl_3$ powder, and (b) XPS spectrum of Ru 3d enlarged from Supplementary Figure 2a.

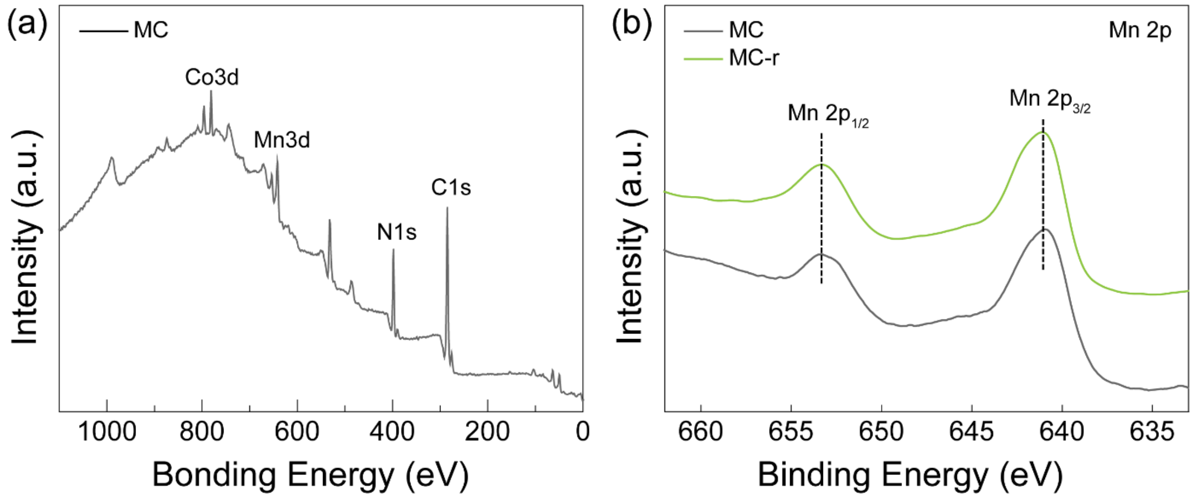

Supplementary Figure 3. XPS characterization of MC and MC-r. (a) XPS spectrum of MC. (b) XPS spectra of Mn 2p from MC and MC-r enlarged in Supplementary Figure 2a and Supplementary Figure 3a, respectively.

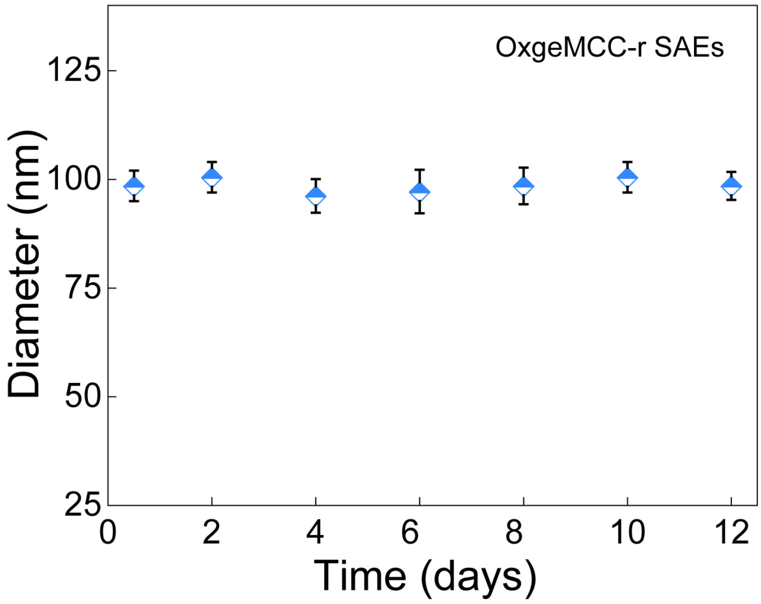

Supplementary Figure 4. Characterization of OxgeMCC-r SAE. Hydrodynamic size distribution of OxgeMCC-r within 12 days. Data are presented as mean ± s.e.m. ($n = 3$).

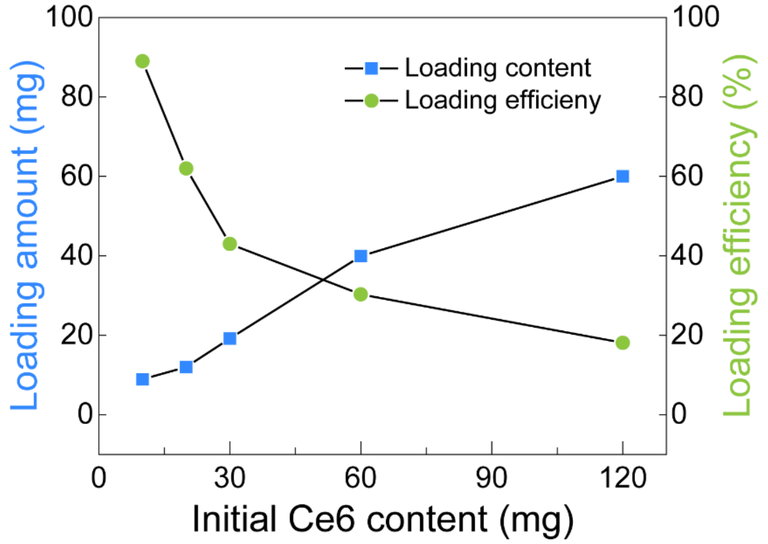

Supplementary Figure 5. Characterization of OxgeMCC-r SAE. Ce6 encapsulation efficiency and loading capacity of OxgeMCC-r under different concentrations of added Ce6.

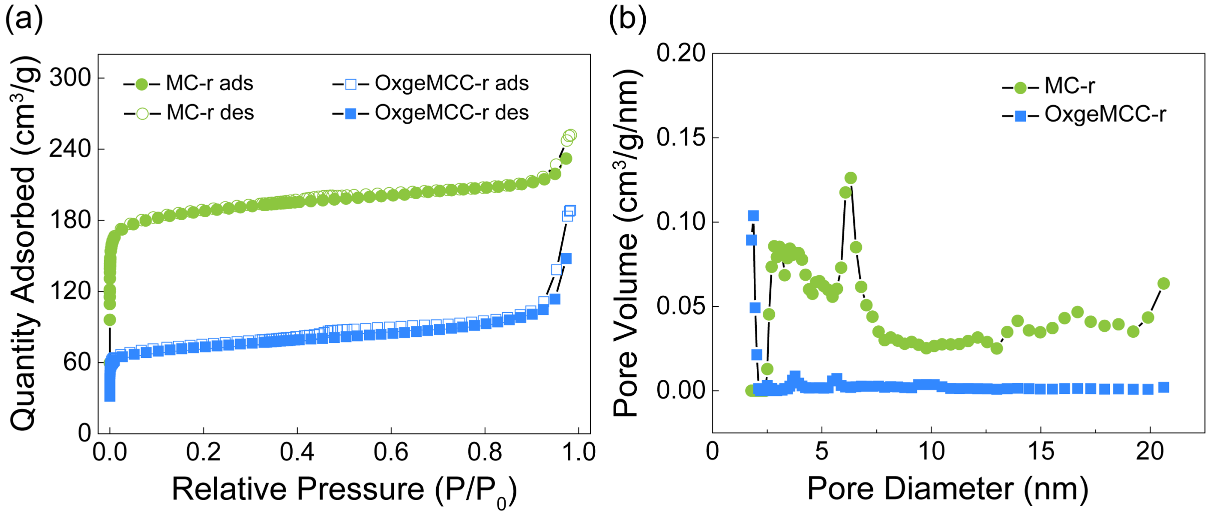

Supplementary Figure 6. **Characterization of MC-r and OxgeMCC-r SAE**. (a) Nitrogen adsorption-desorption isotherms and (b) pore size distributions of MC-r and OxgeMCC-r SAE.

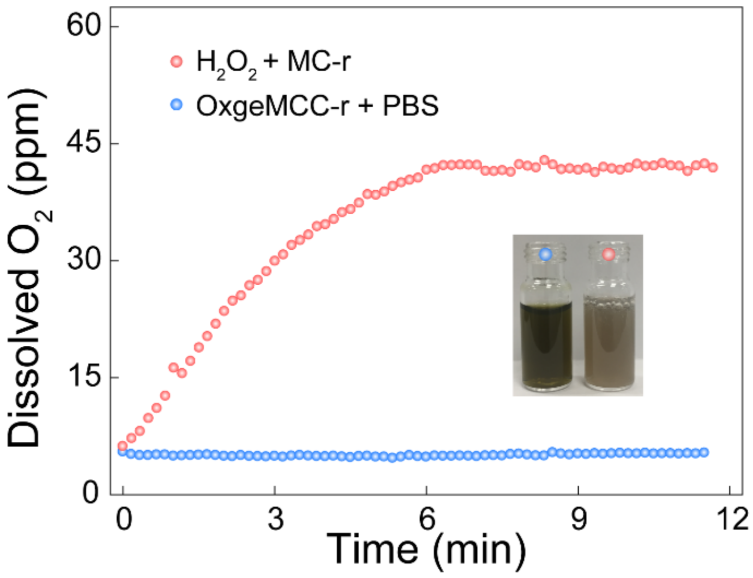

Supplementary Figure 7. **Characterization of MC-r**. O_2 generation after treating with MC-r + H_2O_2 and OxgeMCC-r SAE without H_2O_2 in PBS solution. Inset is a photograph of the two groups.

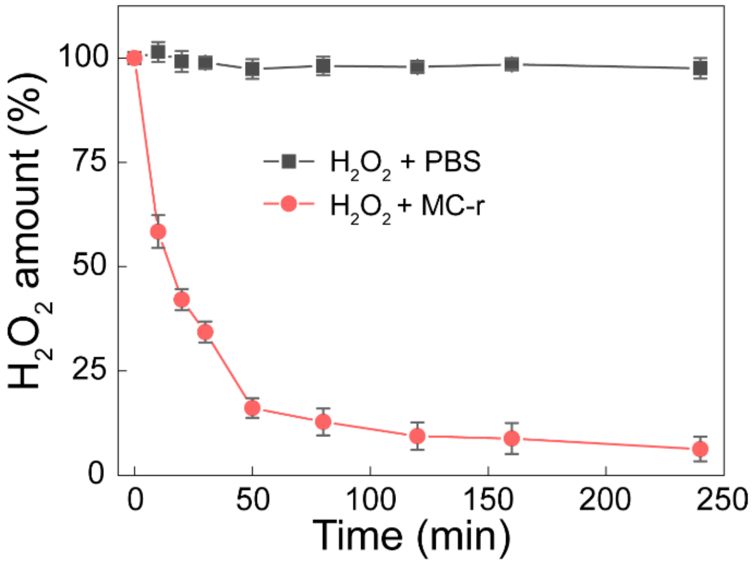

Supplementary Figure 8. **Characterization of MC-r**. Degradation of H_2O_2 in the presence and absence of MC-r in PBS solution. Data are presented as mean ± s.e.m. ($n = 3$).

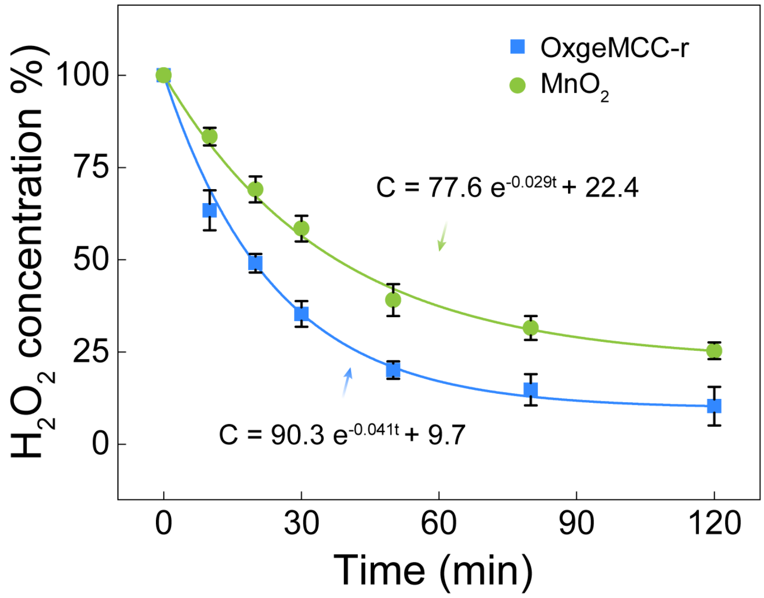

Supplementary Figure 9. **Characterization of MnO_2 and OxgeMCC-r SAE**. Decomposition curve of hydrogen peroxide in the presence of MnO_2 and OxgeMCC-r SAE. Data are presented as mean ± s.e.m. ($n = 3$).

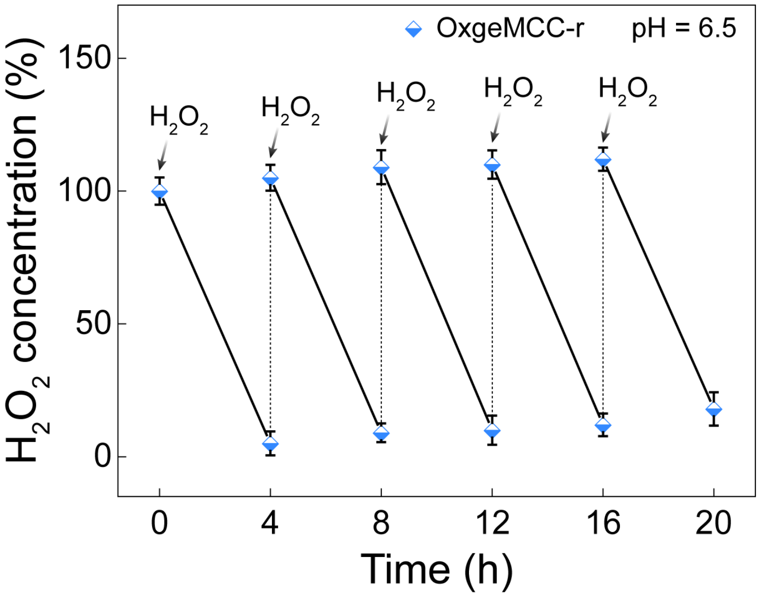

Supplementary Figure 10. **Characterization of OxgeMCC-r SAE**. Repetitive catalytic ability of OxgeMCC-r with repetitive addition of H_2O_2 at pH = 6.5. Data are presented as mean ± s.e.m. ($n = 3$).

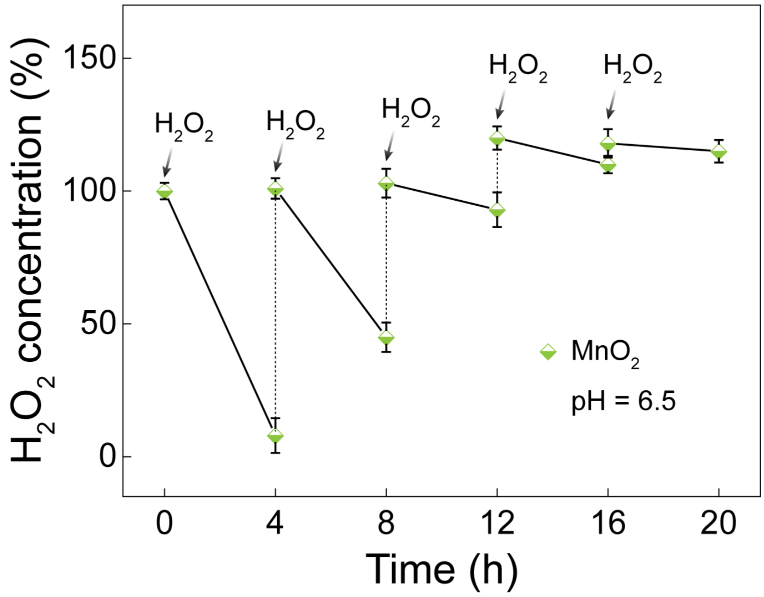

Supplementary Figure 11. **Characterization of MnO_2**. Repetitive catalytic ability of MnO_2 with repetitive addition of H_2O_2 at pH = 6.5. Data are presented as mean ± s.e.m. ($n = 3$).

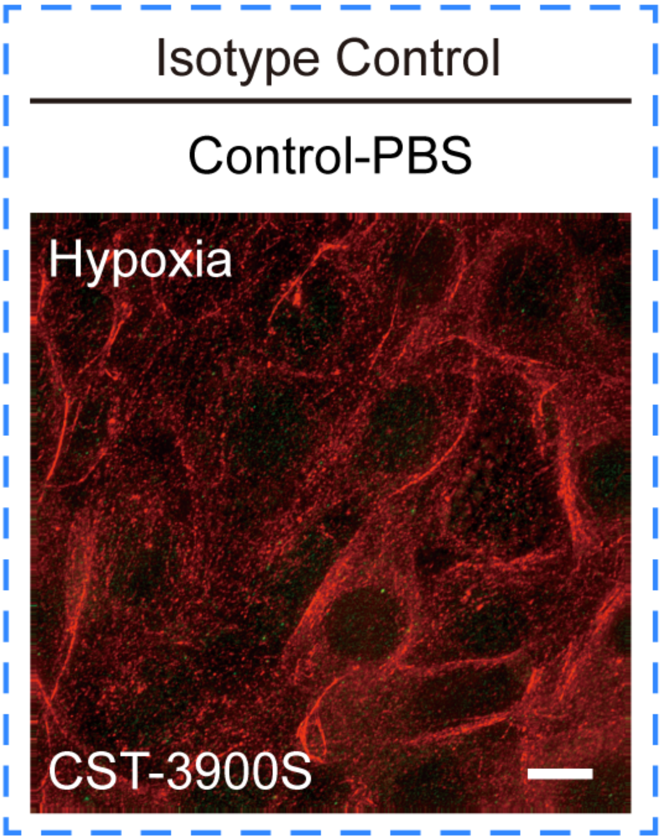

Supplementary Figure 12. **Immunofluorescence imaging of isotype control**. Fluorescence imaging of 4T1 cells with rabbit IgG (CST-3900S, Cell Signaling Technology) isotype control antibody (green) and Tubulin (red) after treated with PBS under hypoxic condition (1% O_2, 5% CO_2, and 94% N_2). Scale bar is 10 μm.

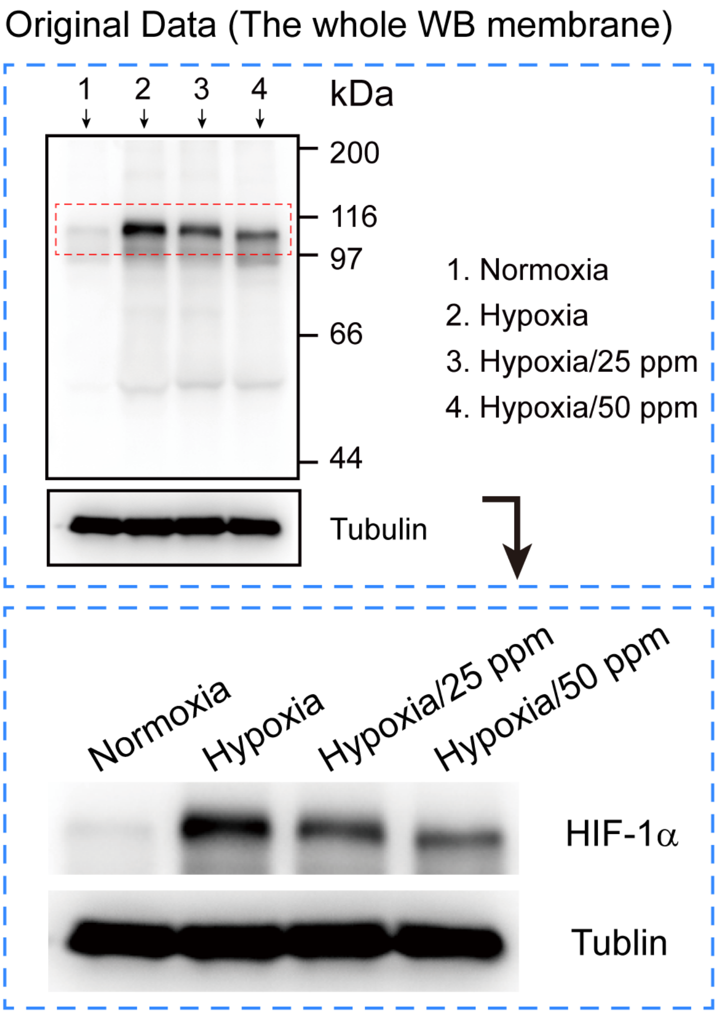

Supplementary Figure 13. **Western blots of HIF-1α**. Original whole WB membrane of HIF-1α expression in 4T1 cells treated with CST-36169T under different conditions.

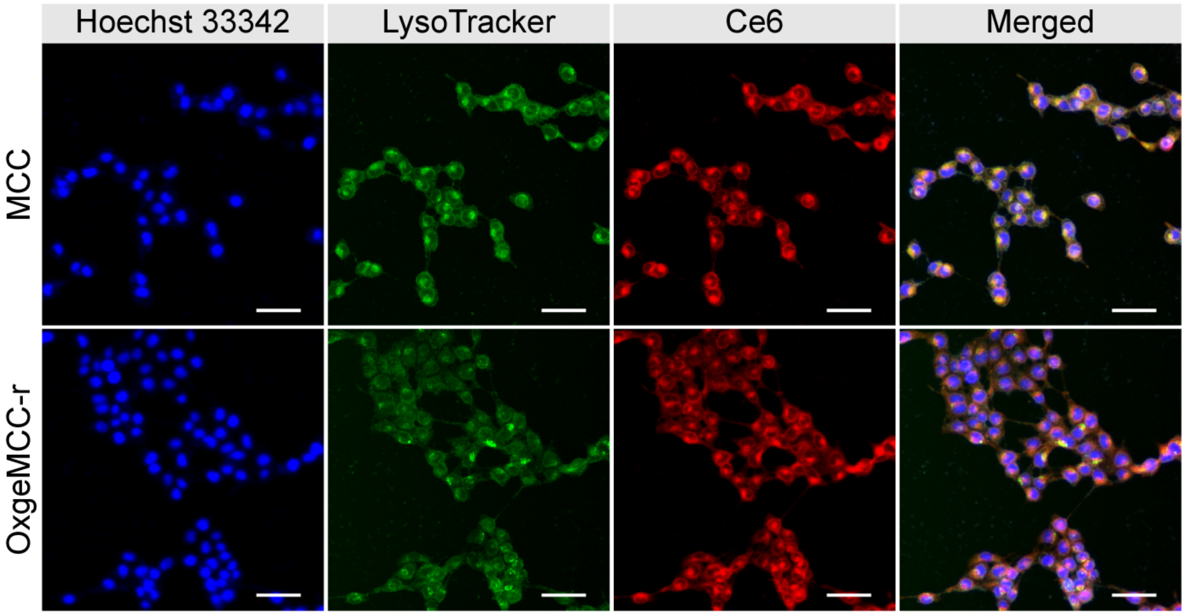

Supplementary Figure 14. **Subcellular localization of MCC and OxgeMCC-r SAE**. Confocal images of MCC and OxgeMCC-r SAEs incubated with 4T1 cells for 4 h. Blue, red, and green colors represent Hoechst 33342, LysoTracker-stained endosomes/lysosomes, and Ce6 fluorescence, respectively. Scale bar = 50 µm.

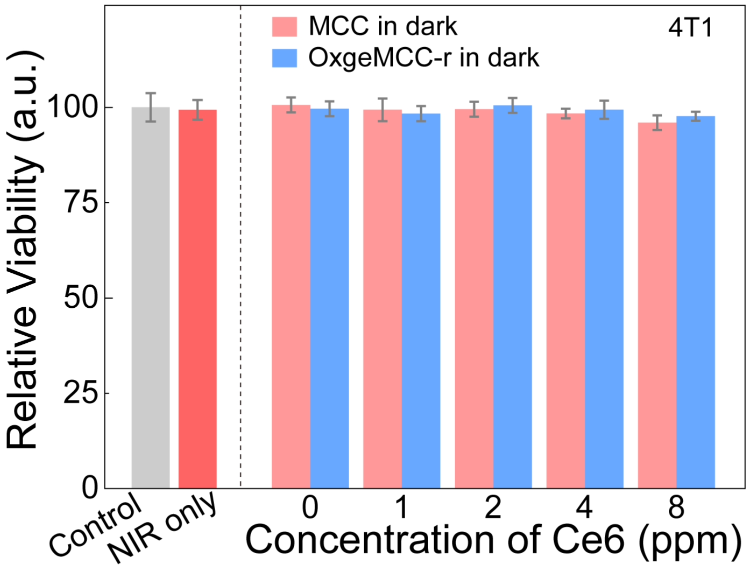

Supplementary Figure 15. **4T1 cell viability under dark condition**. 4T1 cell viability assay of control group, NIR group (671 nm light irradiation), MCC in dark, and OxgeMCC-r SAE in dark at different concentrations. The concentration is based on Ce6. 671 nm laser power density: 100 mW cm^{-2}; irradiation time: 30 seconds. Data are presented as mean ± s.e.m. ($n = 4$).

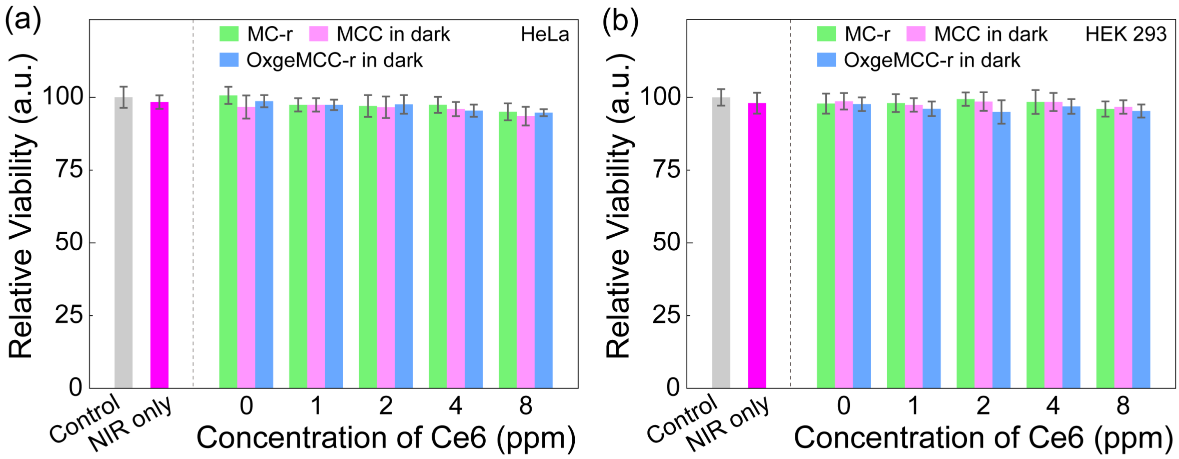

Supplementary Figure 16. **HeLa and HEK 293 cell viability under dark condition**. (a) HeLa and (b) HEK 293 cell viability assay of control group, NIR group (671 nm light irradiation), MC-r, MCC in dark, and OxgeMCC-r SAE in dark at different concentrations. The concentration is based on Ce6. 671 nm laser power density: 100 mW/cm^2; irradiation time: 30 seconds. Data are presented as mean ± s.e.m. ($n = 4$).

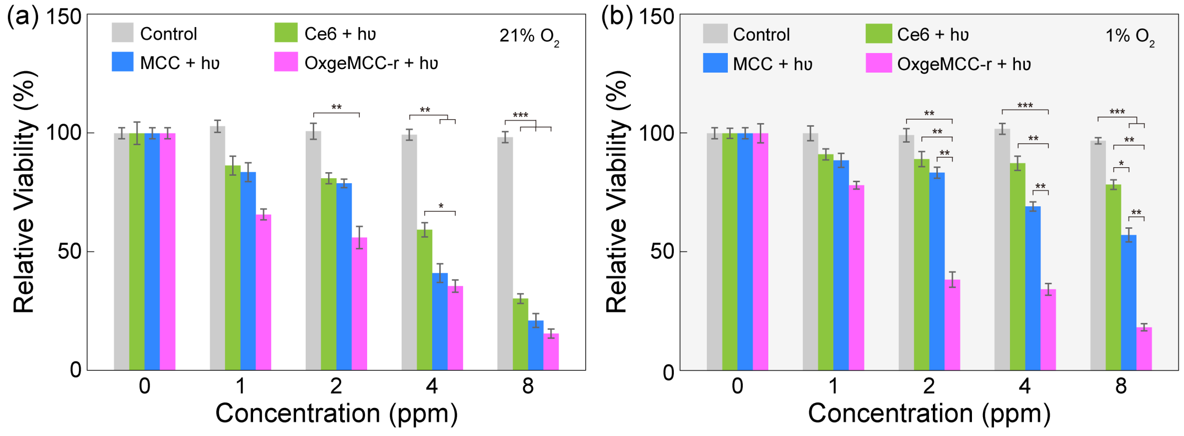

Supplementary Figure 17. ***In vitro* PDT on 4T1 cells using CellTiter-Fluor Cell Viability Assay**. Relative cell viability using CellTiter-Fluor assay for free Ce6, MCC, and OxgeMCC-r SAE treated 4T1 cells in (**a**) normoxic (21% O_2) and (**b**) hypoxic (1% O_2) conditions under 671 nm light irradiation (concentration of Ce6: 8 ppm; 671 nm laser power density: 100 mW cm^{-2}; irradiation time: 30 seconds). Data are presented as mean ± s.e.m. ($n = 4$). Statistical analysis was performed via one-way ANOVA. *$p < 0.05$, **$p < 0.01$, ***$p < 0.001$.

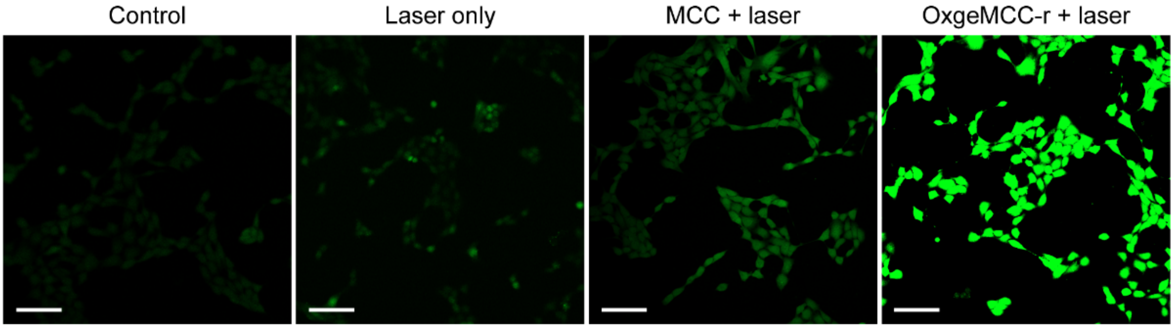

Supplementary Figure 18. **Intracellular ROS staining**. ROS staining in 4T1 cells with indicated treatments in hypoxia condition. Scale bar = 100 μm.

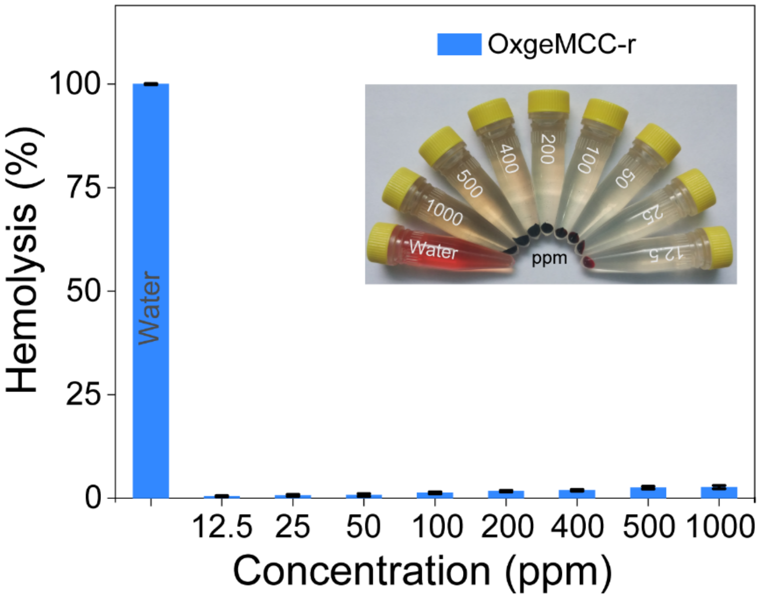

Supplementary Figure 19. **Characterization of OxgeMCC-r SAE**. Hemolysis of OxgeMCC-r solution at various concentrations. Inset: the mixtures were centrifuged to detect the presence of hemoglobin in the supernatants visually. Data are presented as mean ± s.e.m. ($n = 3$).

Supplementary Table 2. Aminotransferase levels at days 8 and 16 relative to the control group. Data are presented as mean ± s.e.m. ($n = 3$).

	**AST (U/L)**	**ALP (U/L)**	**ALT (U/L)**	**BUN (mg/L)**
Reference	55-352	108-367	40-170	70-310
Control	138.1 ± 8.3	174.38 ± 12.3	95.98 ± 6.9	189.74 ± 10.2
8 days	122.85 ± 14.1	180.51 ± 8.4	99.23 ± 9.3	172.16 ± 9.3
16 days	139.42 ± 12.2	177.85 ± 10.7	85.36 ± 14.2	178.02 ± 11.5

Reference ranges of hematology data for healthy female Balb/c mice were obtained from Charles River Laboratories.

Supplementary Table 3. Blood routine analysis results. Data are presented as mean ± s.e.m. ($n = 3$).

	**RBC (M/μL)**	**WBC (K/μL)**	**HGB (g/L)**	**HCT (%)**
Reference	8.16-11.69	5.69-14.84	124-189	43-67
Control	10.19 ± 0.98	6.59 ± 0.45	144.4 ± 11.2	54.1 ± 1.5
8 days	11.76 ± 0.78	7.64 ± 0.32	135 ± 9.2	49.8 ± 2.3
16 days	9.89 ± 0.56	6.52 ± 0.28	154 ± 8.9	52.6 ± 3.4
	**PLT (K/μL)**	**MCV (fL)**	**MPV (fL)**	**MCH (pg)**
Reference	476-1611	50.8-64.1	4.6-5.8	13-17.6
Control	1022 ± 89	55.3 ± 5.3	5.6 ± 0.52	14.06 ± 1.2
8 days	1234 ± 52	52.9 ± 4.2	5.5 ± 0.36	16.33 ± 2.1
16 days	1122 ± 36	56.8 ± 3.7	4.9 ± 0.23	15.56 ± 1.1
	**MCHC (g/L)**			
Reference	239-331			
Control	295 ± 10.5			
8 days	303 ± 9.2			
16 days	312 ± 18.3			

Reference ranges of hematology data for healthy female Balb/c mice were obtained from Charles River Laboratories.

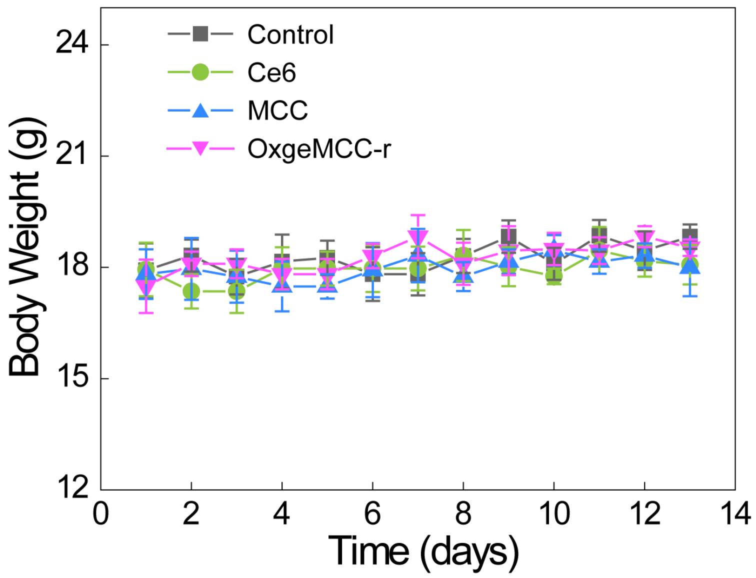

Supplementary Figure 20. **Body weight of mice**. Body weight of mice from different groups during the whole treatments. Data are presented as mean ± s.e.m. ($n = 5$).
