# Supplementary material for: Self-assembled single-atom nanozyme for enhanced photodynamic therapy treatment of tumor
Source: Nat Commun. 2020 Jan 17;11:357. doi: 10.1038/s41467-019-14199-7 (PMC6969186; doi:10.1038/s41467-019-14199-7)
Supplement: Supplementary file 2 — Peer Review [file 41467_2019_14199_MOESM2_ESM.pdf]

Reviewers' comments:

Reviewer #1 (Remarks to the Author); expert in O2 catalysts and PDT, nanotechnology:

In this manuscript, the authors prepared a type of self-assembled single-atom nanozyme, which showed efficient catalase-like property and could significantly enhance the therapeutic efficacy against the hypoxic tumor cells. This work would be of great significance to the development of innovative cancer theranostics for efficient cancer treatment. Therefore, I would like to recommend this work to be accepted for publication in Nature Communication after the following several issues are addressed.
Comments:

1. Based on the Figure 2a and the description in the section of materials, NaBH4 was used for the preparation of nanoformulations of MC-r and OxgenMCC-r. However, the authors did not provide any information about why NaBH4 was used in the synthesis of such MC-r and OxgenMCC-r in the first paragraph of the result section. Therefore, I would like to strongly suggest the authors to clearly describe the reason for the utilization of NaBH4.

2. In figure 4d-f, I think that more controls groups should be included for evaluating the catalytic performance of such OxgeMCC-r SAE.

3. In the control group of Figure 8d, the hypoxia signals are homogeneously distributed through the whole field of vision. However, for typical anti-pimonidazole antibody staining, the hypoxia signals were distant from blood vessels (http://www.hypoxyprobe.com/). Please double check.

4. It seems that the tumor growth curve shown in Figure 8a is not fully consistent with the tumor graph shown in Figure 8b. Please double check.

5. There are a number of typos in the materials section. Please carefully proof-read the manuscript.

Reviewer #2 (Remarks to the Author); expert in polymers trapping gas:

With metal-organic frameworks (MOFs) as the template, the authors designed and synthesized a safe and versatile self-assembled single-atom enzyme (OxgeMCC-r SAE) that consists of catalytically active single-atom ruthenium site for continuous in situ intracellular O2 generation. Advantages of OxgenMCC-r SAE include high Ce6 loading capacity due to intrinsic porous property of the MOF, high catalytic ability, and high catalytic durability for rapid O2 generation from endogenous H2O2 without being self-consumed or requiring external activation. In addition, HAADF-STEM and NEXAFS measurements were performed to study the structure thoroughly. The results have demonstrated that OxgeMCC-r is a remarkable photodynamic agent for cancer therapy together with T1-type MRI ability for in vivo tracking.

This manuscript presents new and intriguing results based on self-assembled single-atom enzyme. The work provides not only an important modality in the area of catalysis-based cancer therapy, but also a guide to other fields such as materials science, chemical engineering, nanotechnology and so on. Thus, it will be of broad interests. Therefore, I strongly recommend its publication in Nature Communications after addressing some minor revisions shown below.

1. "…low toxicity has still be sought after by scientists" should be low toxicity has still been sought after by scientists" in page 3.
2. "… an post-exchange reaction.." should be "… a post-exchange reaction.." in page 4.
3. In page 5, what does "SEAs" refer to?
4. "OcgeMCC-r" should be "OxgeMCC-r" in Figure 4f. What is the unit in Figure 4f?
5. What is the cell viability of OxgeMCC-r in the dark?

6. The Y-axis annotation in Figure 7g is confusing, the authors should confirm this.
7. In Figure 6c, the significance level between OxgeMCC-r + hv and Ce6 + hv was not indicated. In Figure 5c, the significance level of normoxia and hypoxia/50 ppm was also not indicated.

Reviewer #3 (Remarks to the Author); expert in Photodynamics and cancer therapy:

The manuscript describes the synthesis of novel nanoagents (NA) that catalyze oxygen generation directly in cancer cells to alleviate hypoxia. Moreover, the NA were loaded with a photosensitizer, then used in photodynamic therapy (PDT) of cancer and MRI diagnosis. This approach is novel and interesting since PDT therapeutic efficiency is often limited by local oxygen concentration. The nanoplatform also shows some potential for further improvements i.e. loading with NIR-activated photosensitizers or targeting cancer associated antigens using antibodies. However, there are several issues that need to be addressed:

1) MTT assay is not a good choice for a viability assay since it utilizes mitochondria to produce formazan. Mitochondrial activity is significantly limited following exposure of cancer cells to hypoxia. The second independent assay, that is not affected by the metabolic state of a cell, should be used to determine cell viability (e.g. CellTox Green). Moreover the authors used MC-r nanoparticles to study cell viability but used MCC-r and OxgeMCC-r for in vivo studies (Figure 5a). It would be interesting to determine how MCC-r and OxgeMCC-r affect cytotoxicity/proliferation of additional cancer cell lines (e.g. gliomas) and at least one non-cancerous cell line.

2) Figure 4f is not clear. Is should clearly marked which bars represent normoxic/hypoxic conditions. Did the authors use only one control for both normoxia/hypoxia? There is one typo: "OcgeMCC-r".

3) WB and confocal images targeting HIF-1aplha are not convincing. The authors used anti-HIF-1 alpha antibody ab190197 (IHC) or ab51608 (WB). Both Abs target human HIF-1alpha but mouse cell lines were used. Human HIF-1 alpha aa 600-700 was used as an immunogen by Abcam. The homology of this fragment between human and mouse is approx. 66%. For mouse specific Hif-1-alpha the company recommends the ab179483. Moreover it seems that the authors did not use isotype control for IHC studies (e.g. ab199091). HIF-1 alpha is a difficult target since it degrades rapidly in the presence of oxygen. The authors should prove that the ab190197 and ab51608 Abs targeted mouse HIF-1alpha. I would like to see the whole WB membrane, not only a small fragment.

4) Materials and methods: I presume that Ab used for WB was ab51608 but not ab190197.

5) Please provide more information about the light source and add the light doses for each experiment (J/cm^2) not only fluence rate and time.

6)Figure 4: irradiation 100 mW/cm^2, 30 seconds.; Materials and methods-singlet oxygen detection: 100 mW/cm^2, 30 min.?

7)Figure 6: lack of the light control and dark control. See also the point 1.

8) What is the subcellular localization of MCC-r and OxgeMCC-r?

9) For more than two samples ANOVA (e.g. Fig. 5c/6a/6b/8c) or ANCOVA (Fig.8a) needs to be used to analyse the statistical significance.

Response to Reviewers' Comments:

For Reviewer #1: *(Remarks to the Author); expert in O_2 catalysts and PDT, nanotechnology:*

Comments: *In this manuscript, the authors prepared a type of self-assembled single-atom nanozyme, which showed efficient catalase-like property and could significantly enhance the therapeutic efficacy against the hypoxic tumor cells. This work would be of great significance to the development of innovative cancer theranostics for efficient cancer treatment. Therefore, I would like to recommend this work to be accepted for publication in Nature Communication after the following several issues are addressed.*

Re: We really appreciate the reviewer's useful comments and positive recommendation of publication after revisions. We have carefully revised the manuscript based on the comments.

1. Based on the Figure 2a and the description in the section of materials, $NaBH_4$ was used for the preparation of nanoformulations of MC-r and OxgenMCC-r. However, the authors did not provide any information about why $NaBH_4$ was used in the synthesis of such MC-r and OxgenMCC-r in the first paragraph of the result section. Therefore, I would like to strongly suggest the authors to clearly describe the reason for the utilization of $NaBH_4$.

Re: Thank you for the useful question! Metal-organic frameworks (MOFs) can be suitable candidates for constructing single-atom nanomaterials on account of their well-defined coordination network. Previous work showed that, under reduced hydrothermal conditions, Prussian blue analogue $Mn_3[Fe(CN)_6]_2$ can be used as a substrate for constructing Pd and Pt single-atom nanocatalysts, in which Pd and Pt took the places of partial Fe nodes (Ref. 37). Herein, using another Prussian blue analogue $Mn_3[Co(CN)_6]_2$ as the substrate, the Ru ion firstly took the place of Co nodes within the framework (Ref. 38). As a mimetic reduction environment, mild reduction microenvironment was created with low concentration $NaBH_4$ as the reduction agent (Ref. 48,49). Under this mild reduction condition, the Ru node can be anchored firmly through both the coordination interaction and reduction in the MOFs. Related discussions have been added in the revised manuscript (**Page 4, paragraph 2 and Page 5, paragraph 2**).

2. In figure 4d-f, I think that more controls groups should be included for evaluating the catalytic performance of such OxgeMCC-r SAE.

Re: Thanks for this recommendation. We have added other control groups including MC-r group in **Figure 4d**,**e**. As shown in **Figures R1** and **R2**, new results indicate that, only with the formation of single-atom Ru within the structure of MOFs, the obtained nanozymes could behave catalase-like property for boosted oxygen generation. **Figures R1** and **Figure R2** have been added in the revised supporting file as **Figures S7** and **S8**, and corresponding descriptions have been added in the revised manuscript **(Page 6, paragraph 2**). In addition, we have also

added one control group under hypoxic condition for **Figure 4f**.

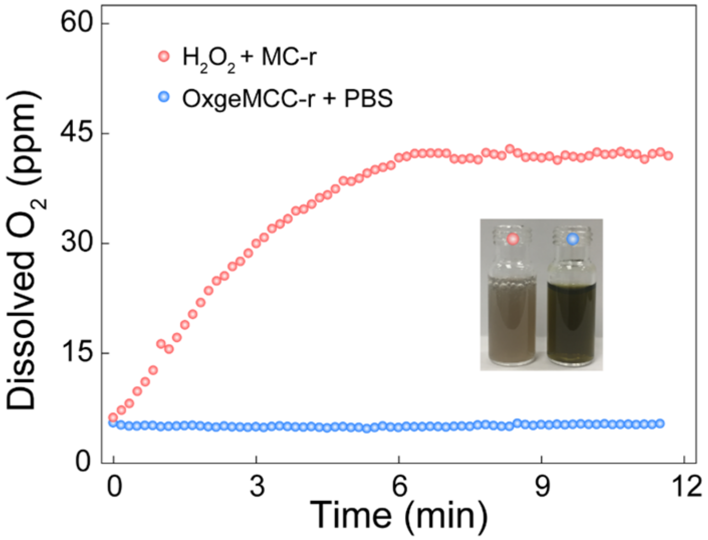

Figure R1. O_2 generation after treating with MC-r + H_2O_2 and OxgeMCC-r SAE without H_2O_2 in PBS solution. Inset is a photograph of two groups.

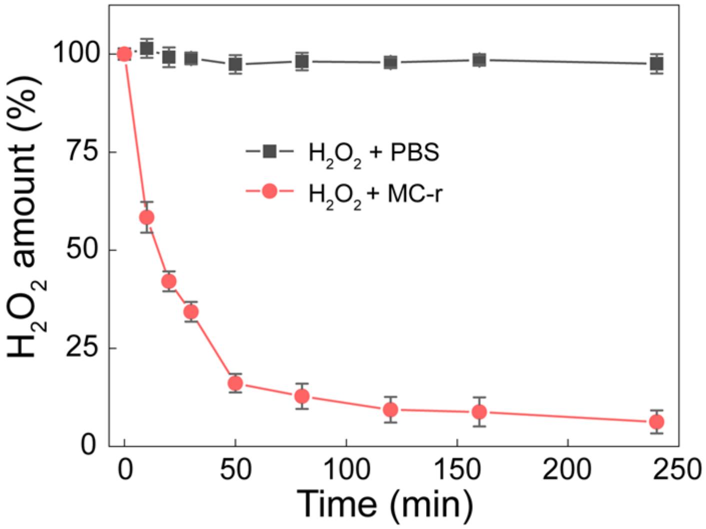

Figure R2. Degradation of H_2O_2 in the presence and absence of MC-r in PBS solution.

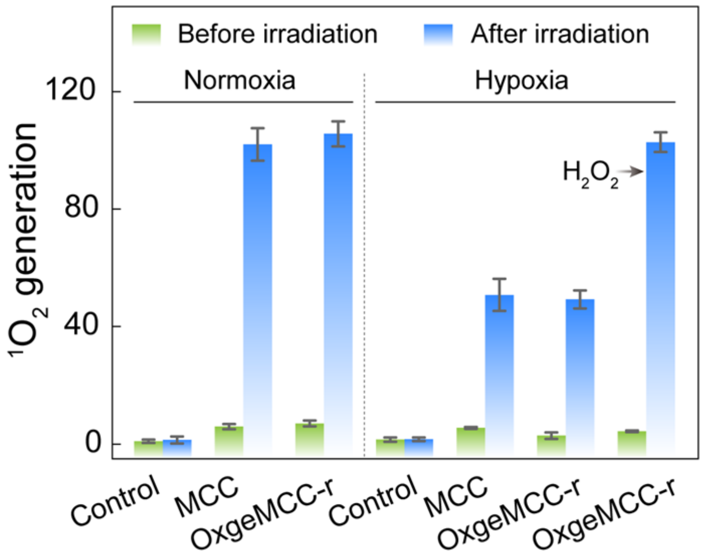

Figure 4f. Singlet oxygen ($^1O_2$) generation efficiency determined by DPBF under indicated conditions before and after 671 nm laser irradiation (100 mW cm^{-2}, 30 seconds).

3. In the control group of Figure 8d, the hypoxia signals are homogeneously distributed through the whole field of vision. However, for typical anti-pimonidazole antibody staining, the hypoxia signals were distant from blood vessels (http://www.hypoxyprobe.com/). Please double check.

Re: Thank you for the reminder! For the previous data, the antigen-antibody specificity or the experiment process may affect the results. For this reason, we have re-performed the immunofluorescence experiments more carefully according to the supplier protocol. For the untreated group, it is no doubt that the hypoxia signals were distant from the blood regions. For the regions without blood vessels, hypoxia positive is shown. For the OxgeMCC-r treated group, the hypoxia condition is significantly ameliorated. Herein, it should be noted that even in the some area, positive signals of blood vessels could be observed. Sometimes, those blood vessels are closed. In this way, those blood vessels would lose the ability to transport molecular oxygen. **Figure 8d** has been updated in the revised manuscript.

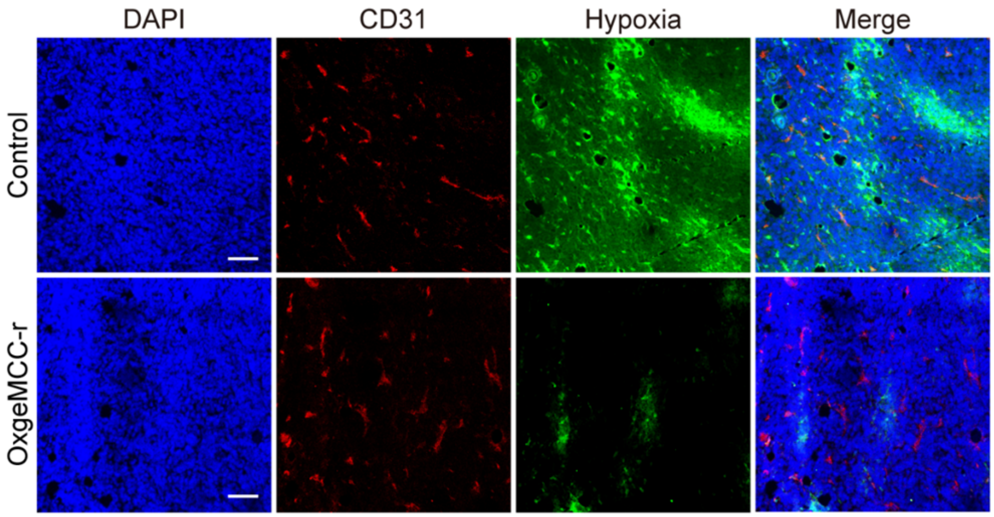

Figure 8d. Representative hypoxia immunofluorescence images of tumor slices. Nuclei, blood vessels, and hypoxic regions were stained by 4',6-diamidino-2-phenylindole (blue), anti-CD31 antibody (red), and anti-pimonidazole antibody (green), respectively (three mice for each group). Scale bar is 100 μm.

4. It seems that the tumor growth curve shown in Figure 8a is not fully consistent with the tumor graph shown in Figure 8b. Please double check.

Re: Thank you for your advice! The tumor growth curves were measured by a caliper every other day during the therapy process. Usually, the shape of the tumor is heterogeneous and sometimes the tumor may embed into the muscle regions. Thus, the growth curves may not be fully consistent with the tumor obtained after the anatomy. The tumor weight after anatomy is more accurate.

5. There are a number of typos in the materials section. Please carefully proof-read the manuscript.

Re: Thank you for careful reading. We have thoroughly checked the manuscript to eliminate any language issues.

For Reviewer #2: *(Remarks to the Author); expert in polymers trapping gas:*

Comments: *With metal-organic frameworks (MOFs) as the template, the authors designed and synthesized a safe and versatile self-assembled single-atom enzyme (OxgeMCC-r SAE) that consists of catalytically active single-atom ruthenium site for continuous in situ intracellular O2 generation. Advantages of OxgenMCC-r SAE include high Ce6 loading capacity due to intrinsic porous property of the MOF, high catalytic ability, and high catalytic durability for rapid O2 generation from endogenous H2O2 without being self-consumed or requiring external activation. In addition, HAADF-STEM and NEXAFS measurements were performed to study the structure thoroughly. The results have demonstrated that OxgeMCC-r is a remarkable photodynamic agent for cancer therapy together with T1-type MRI ability for in vivo tracking.*

This manuscript presents new and intriguing results based on self-assembled single-atom enzyme. The work provides not only an important modality in the area of catalysis-based cancer therapy, but also a guide to other fields such as materials science, chemical engineering, nanotechnology and so on. Thus, it will be of broad interests. Therefore, I strongly recommend its publication in Nature Communications after addressing some minor revisions shown below.

Re: We really appreciate the reviewer's useful comments and positive recommendation of publication after minor revisions. All the concerns have been fully addressed in the revised manuscript, as detailed below.

1. "…low toxicity has still be sought after by scientists" should be low toxicity has still been sought after by scientists" in page 3.

Re: We agree! The change has been made as per the advice in the revised manuscript (**Page 3, paragraph 1**).

2. "… an post-exchange reaction.." should be "… a post-exchange reaction.." in page 4.

Re: Thank you! The change has been made as per the advice in the revised manuscript (**Page 4, paragraph 2**).

3. In page 5, what does "SEAs" refer to?

Re: We are sorry for the confusion! This has been revised into "SAEs", which means "single-atom enzymes" in the revised manuscript.

4. "OcgeMCC-r" should be "OxgeMCC-r" in Figure 4f. What is the unit in Figure 4f?

Re: Thank you! "OcgeMCC-r" has been updated into "OxgeMCC-r". The unit in **Figure 4f** is a relative value in which the total degradation of DPBF indicator is defined as 100%.

5. What is the cell viability of OxgeMCC-r in the dark?

Re: Thank you for the advice! The cell viability of both MCC and OxgeMCC-r SAE was performed in dark condition with the same concentrations to *in vitro* therapy. As shown in **Figure R3**, there was no obvious killing ability of OxgeMCC-r SAE for the tested concentrations. Besides, the NIR only group also showed negligible toxicity to the tested 4T1 cells. **Figure R3** has been added in the revised supporting file as **Supplementary Fig. 15**, and corresponding descriptions have been added in the revised manuscript **(Page 8, paragraph 2)**.

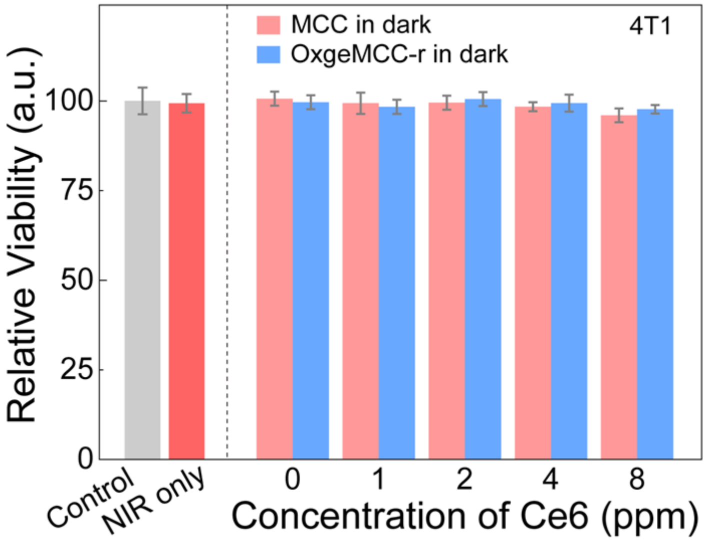

Figure R3. **4T1 cell viability under dark condition**. 4T1 cell viability assay of control group, NIR group (671 nm light irradiation), MCC in dark, and OxgeMCC-r SAE in dark of different concentrations. (The concentration is based on Ce6; 671 nm laser power density: 100 mW cm^{-2}; irradiation time: 30 seconds).

6. The Y-axis annotation in Figure 7g is confusing, the authors should confirm this.

Re: We are sorry for the confusion! It is now corrected in **Figure 7g** of the revised manuscript.

7. In Figure 6c, the significance level between OxgeMCC-r + hv and Ce6 + hv was not indicated. In Figure 5c, the significance level of normoxia and hypoxia/50 ppm was also not indicated.

Re: Thanks! As suggested, related information has been added in the revised manuscript.

For Reviewer #3: *(Remarks to the Author); expert in Photodynamics and cancer therapy:*

Comments: *The manuscript describes the synthesis of novel nanoagents (NA) that catalyze oxygen generation directly in cancer cells to alleviate hypoxia. Moreover, the NA were loaded with a photosensitizer, then used in photodynamic therapy (PDT) of cancer and MRI diagnosis. This approach is novel and interesting since PDT therapeutic efficiency is often limited by local oxygen concentration. The nanoplatform also shows some potential for further improvements i.e. loading with NIR-activated photosensitizers or targeting cancer associated antigens using antibodies. However, there are several issues that need to be addressed:*

Re: We really appreciate the reviewer's useful comments and suggestions. We have carefully revised the manuscript based on the comments.

1. MTT assay is not a good choice for a viability assay since it utilizes mitochondria to produce formazan. Mitochondrial activity is significantly limited following exposure of cancer cells to hypoxia. The second independent assay that is not affected by the metabolic state of a cell, should be used to determine cell viability (e.g. CellTox Green). Moreover the authors used MC-r nanoparticles to study cell viability but used MCC and OxgeMCC-r for in vivo studies (Figure 5a). It would be interesting to determine how MCC and OxgeMCC-r affect cytotoxicity/proliferation of additional cancer cell lines (e.g. gliomas) and at least one non-cancerous cell line.

Re: Thank you for your valuable advice! Upon receiving this comment, we bought the CellTox Green (Promega G8742) from Promega Pte Ltd. This CellTox Green cytotoxicity assay is mitochondrial activity-independent, which measures the membrane integrity. When dyes bond DNA of compromised cells, the fluorescence is substantially enhanced. However, when this assay applied to our experiment, it did not work. All wells including the control groups and experimental groups showed almost no fluorescence. We tried this experiment for almost one month. After consulting the supplier, we noticed an important thing that this assay only works with compromised cells whose membrane is seriously destroyed, and thereafter the dyes can enter the cells to bind the dead cell DNA. In our case, however, the cells after PDT are not necrosis, and the death path is apoptosis (based on the flow cytometry results in **Figure 6d**). Although these cells suffered apoptosis, their membranes are still not seriously destroyed. Then, we carried out another assay named CellTiter-Fluor Cell Viability Assay (Promega G6081). This assay is also mitochondrial activity-independent, which measures the protease activity within live cells. The substrates can easily enter intact cells where the substrates are cleaved by the live-cell protease activity to generate a fluorescent signal proportional to the number of living cells. Thus, this assay was applied on 4T1 cells. As shown in **Figure R4**, the results correlate well with the MTT results. After PDT, the cells died through apoptosis pathway (**Figure 6d**). Although the membrane of the dead cells was not destroyed seriously, the protease activity was already lost. **Figure R4** has been added in the revised supporting file as

Supplementary Fig. 17, and corresponding descriptions have been added in the revised manuscript **(Page 8, paragraph 2**).

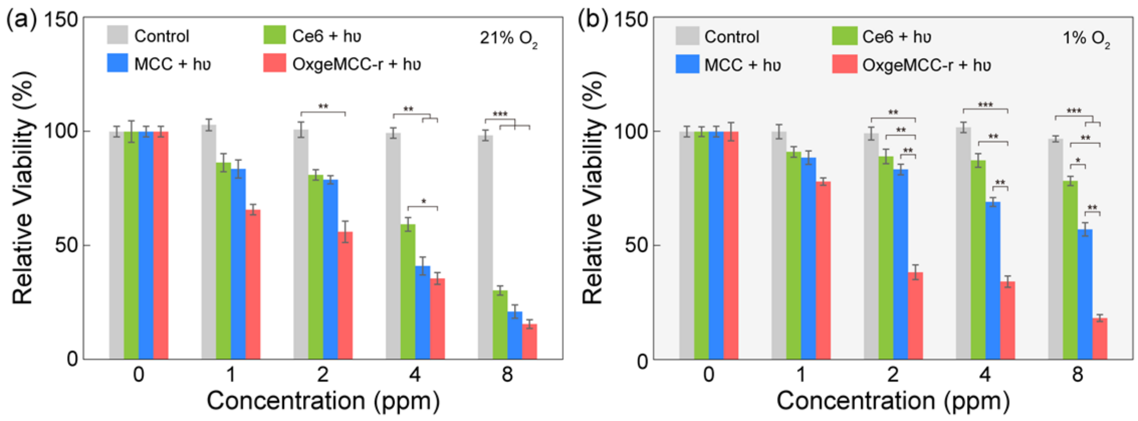

Figure R4. ***In vitro* PDT on 4T1 cells using CellTiter-Fluor Cell Viability Assay**. Relative cell viability using CellTiter-Fluor Cell Viability Assay of free Ce6, MCC, and OxgeMCC-r SAE treated 4T1 cells in (**a**) normoxic and (**b**) hypoxic conditions under 671 nm light irradiation (concentration of Ce6: 8 ppm; 671 nm laser power density: 100 mW cm^{-2}; irradiation time: 30 seconds).

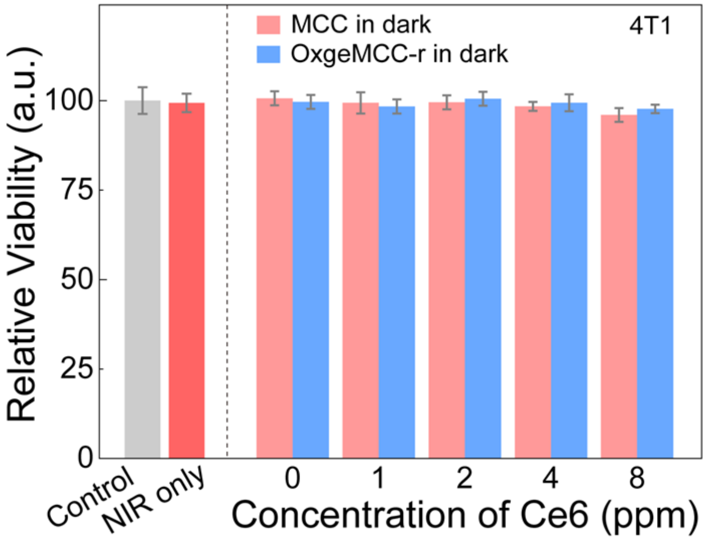

Figure R3. **4T1 cell viability under dark condition**. 4T1 cell viability assay of control group, NIR group (671 nm light irradiation), MCC in dark, and OxgeMCC-r SAE in dark of different concentrations. (The concentration is based on Ce6; 671 nm laser power density: 100 mW cm^{-2}; irradiation time: 30 seconds).

In addition, we have also tested the cytotoxicity of MC-r, MCC and OxgeMCC-r SAE on 4T1 cells, HeLa cells, and non-cancerous cells (HEK 293, Human embryonic kidney 293) at the same concentrations to those for *in vitro* PDT. As shown in **Figures R3** and **R5**, the results also

indicated the negligible cytotoxicity of MC-r to all tested cell lines. Both MCC and OxgeMCC-r SAE showed no killing efficiency under the dark condition. **Figure R3** has been added in the revised supporting file as **Supplementary Fig. 15**, and corresponding descriptions have been added in the revised manuscript **(Page 8, paragraph 2**). **Figure R5** has been added in the revised supporting file as **Supplementary Fig. 16**, and corresponding descriptions have been added in the revised manuscript **(Page 8, paragraph 2**).

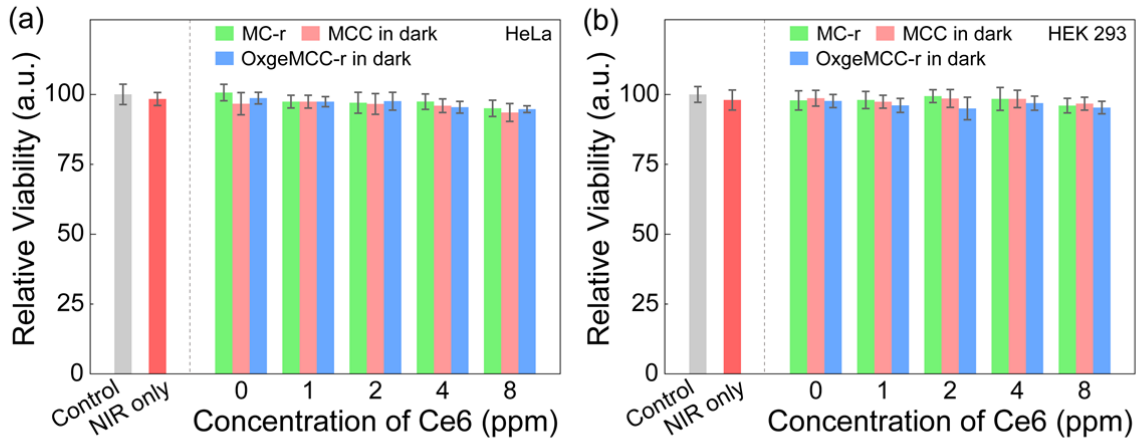

Figure R5. **HeLa and HEK 293 cells viability under dark condition**. (a) HeLa and (b) HEK 293 cell viability assay of control group, NIR group (671 nm light irradiation), MC-r, MCC in dark, and OxgeMCC-r SAE in dark of different concentrations. (The concentration is based on Ce6; 671 nm laser power density: 100 mW/cm^2; irradiation time: 30 seconds).

2. Figure 4f is not clear. Is should clearly marked which bars represent normoxic/hypoxic conditions. Did the authors use only one control for both normoxia/hypoxia? There is one typo: "OcgeMCC-r".

Re: Thank you for the advice! As suggested, we have marked clear indicators for normoxic/hypoxic conditions. Another control group in hypoxic condition has also been added. "OcgeMCC-r" has been updated to "OxgeMCC-r". We have revised these in **Figure 4f** of the revised manuscript.

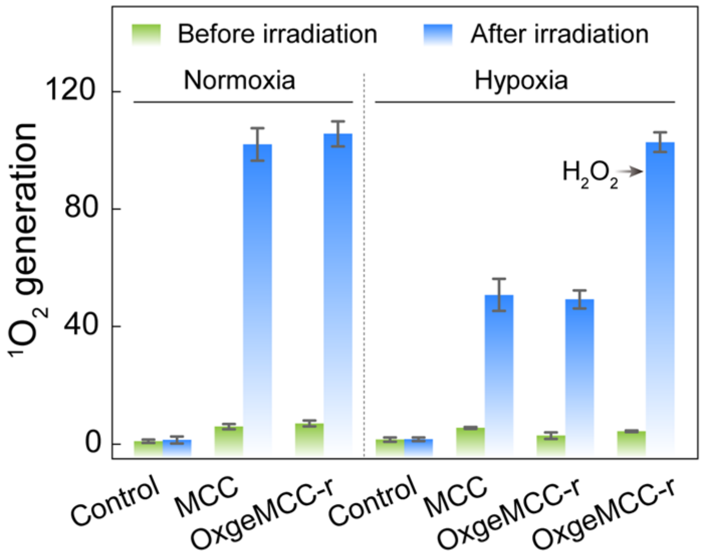

Figure 4f. Singlet oxygen ($^1O_2$) generation efficiency determined by DPBF under indicated conditions before and after 671 nm laser irradiation (100 mW cm^{-2}, 30 seconds).

3. WB and confocal images targeting HIF-1aplha are not convincing. The authors used anti-HIF-1 alpha antibody ab190197 (IHC) or ab51608 (WB). Both Abs target human HIF-1alpha but mouse cell lines were used. Human HIF-1 alpha aa 600-700 was used as an immunogen by Abcam. The homology of this fragment between human and mouse is approx. 66%. For mouse specific HIF-1-alpha the company recommends the ab179483. Moreover it seems that the authors did not use isotype control for IHC studies (e.g. ab199091). HIF-1 alpha is a difficult target since it degrades rapidly in the presence of oxygen. The authors should prove that the ab190197 and ab51608 Abs targeted mouse HIF-1alpha. I would like to see the whole WB membrane, not only a small fragment.

Re: We sincerely thank the reviewer for the professional suggestion! After receiving the comments, we first tried the suggested Cat#ab179483 product (Batch No: GR225901-1) from Abcam, as it is a mouse specific anti-HIF-1α antibody. However, as shown in **Figure R6**, results did not show the targeted protein at the right position. At this situation, we selected the human specific anti-HIF-1α antibody (Cat#ab51608, Batch No: GR244245-43). Results indicated the targeted protein at the right position. With the increase of incubation concentration of OxgeMCC-r SAEs, the level of HIF-1α protein is down regulated. Just as the reviewer mentioned, the homology of the fragment between human and mouse is approx. 66%. During the test, we used a relatively higher antibody concentration (about 1.049-1.168 mg/mL) for WB. According to the protocol provided by the supplier, the suggested dilution is 1:100 for WB. Thus, the higher Ab concentration should be the reason for our previous results in **Figure R7** (The whole WB membrane in Figure 5f of our previous version).

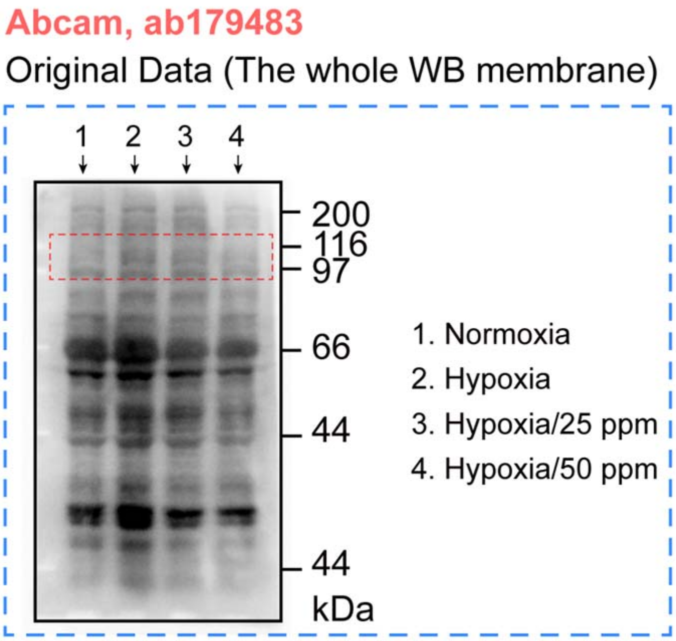

Figure R6. **Western blots of HIF-1α**. Original whole WB membrane of HIF-1α expression in 4T1 cells treated under different conditions with ab179483.

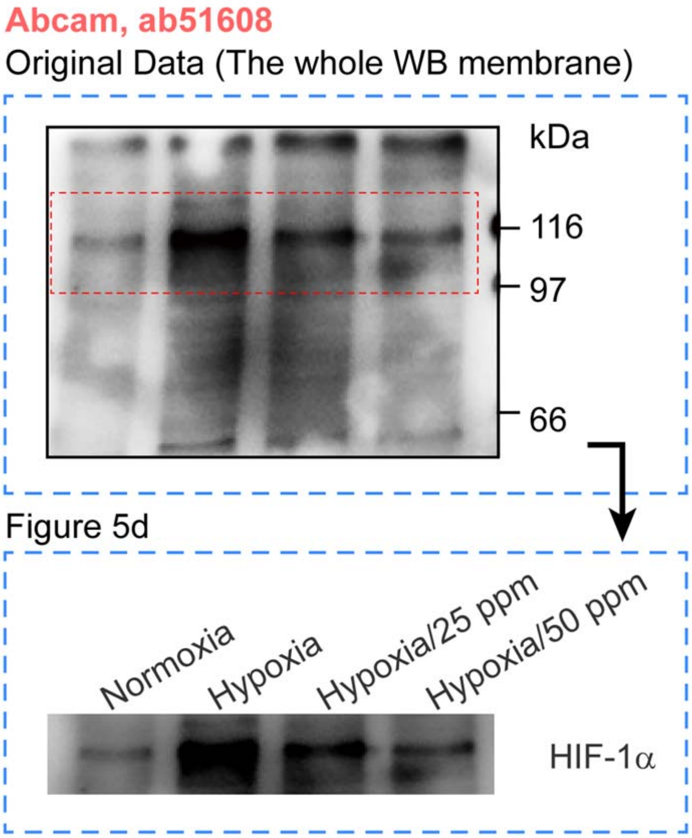

Figure R7. **Western blots of HIF-1α**. Original whole WB membrane of HIF-1α expression in 4T1 cells treated under different conditions with ab51608.

As the product Cat#ab179483 for mouse-specific anti-HIF-1α did not work well, we brought and tested another product form Cell Signaling Technology (Cat#CST-36169T), which is also a mouse-specific anti-HIF-1α antibody. As shown in the original whole WB membrane (**Figure R8**), it displayed the consistent trend compared with the data gained from Cat#ab51608. Thus, the data gained from our previously used Cat#ab51608 are convincing. Thus, we recommend the product (Cat#CST-36169T) from Cell Signaling Technology for such research. **Figure R8** has been added in the revised supporting file as **Supplementary Fig. 13**, and corresponding descriptions have been added in the revised manuscript **(Page 7, paragraph 2**).

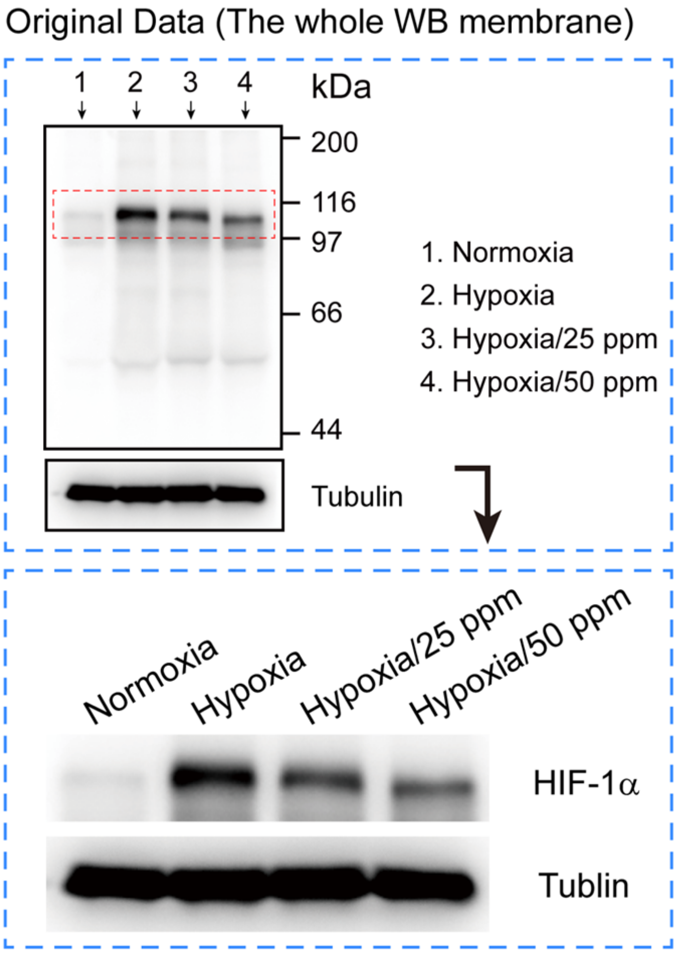

Figure R8. **Western blots of HIF-1α**. Original whole WB membrane of HIF-1α expression in 4T1 cells treated under different conditions with CST-36169T.

For the immunofluorescence test, we re-performed it again with the product from Cell Signaling Technology (Cat#CST-36169T) instead of anti-HIF-1α Ab from Abcam (Cat#ab190197). As shown in **Figure 5b**, the immunofluorescence imaging results showed no obvious expression of HIF-1α under normoxia condition. In contrast, when incubated under hypoxia condition, enhanced expression of HIF-1α was observed with the brightest green fluorescence. Upon treating with MC-r from 25 to 50 ppm, the expression of HIF-1α was

significantly down regulated, suggesting that efficient oxygenation induced by MC-r attenuated the hypoxic condition. It is notable that the results using anti-HIF-1α Ab from Cell Signaling Technology (Cat#CST-36169T) are consistent with that acquired from Abcam (Cat#ab190197) in our previous version. Furthermore, we also performed the isotype control using Rabbit (DA1E) mAb IgG XP® Isotype Control (Cat#CST-3900S) to exclude the non-specific binding. The concentration of the isotype control antibody is the same as the specific anti-HIF-1α Ab used. As shown in **Figure R9**, results showed almost no green fluorescence for the isotype control. **Figure R9** has been added in the revised supporting file as **Supplementary Fig. 12**, and corresponding descriptions have been added in the revised manuscript **(Page 7, paragraph 2)**.

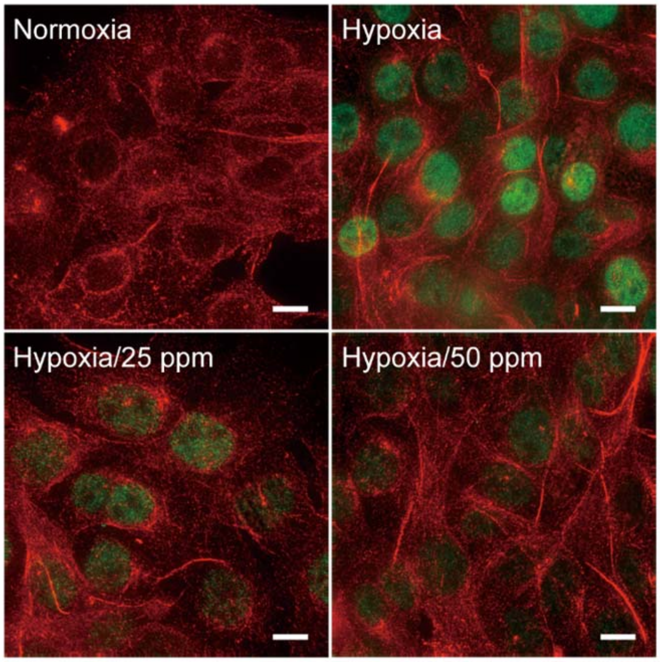

Figure 5b. Fluorescence imaging of 4T1 cells with stained HIF-1α (green) and Tubulin (red) after treated with PBS under normoxic condition (21% O_2, 5% CO_2, and 74% N_2) as well as PBS (Hypoxia) and MC-r (Hypoxia/25 ppm and Hypoxia/50 ppm) under hypoxic condition (1% O_2, 5% CO_2, and 94% N_2). Scale bar is 10 μm.

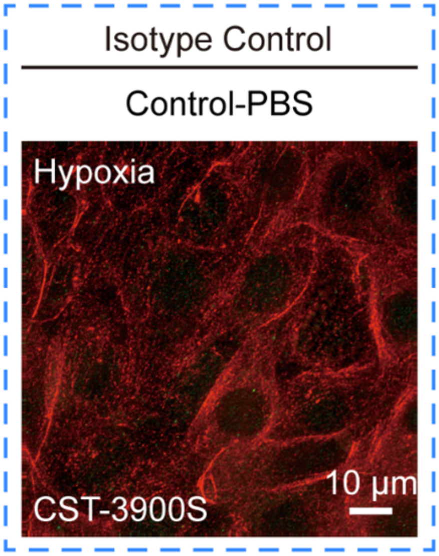

Figure R9. **Immunofluorescence imaging of isotype control**. Fluorescence imaging of 4T1 cells with rabbit IgG (CST-3900S, Cell Signaling Technology) isotype control antibody (green) and Tubulin (red) after treated with PBS under hypoxic condition (1% O_2, 5% CO_2, and 94% N_2). Scale bar is 10 μm.

4. Materials and methods: I presume that Ab used for WB was ab51608 but not ab190197.

Re: We agree! The Ab used for WB in our previous manuscript is ab51608. In the revised manuscript, we used the product CST-36169T (HIF-1α (D1S7W) XP® Rabbit mAb) from Cell Signaling Technology for WB.

5. Please provide more information about the light source and add the light doses for each experiment (J/cm^2) not only fluence rate and time.

Re: Thanks for the reminder. For our experiments, the power density is 100 mW/cm^2, and the area of the laser spot in wells or on mice is 1 cm^2. As we know, Watt = Joules per Second, so 100 mW is 100 mJ/s or 0.1 J/s. During 1 second, the light dose is 0.1 J/cm^2. This information has been added in the "Materials and Methods" section.

6. Figure 4: irradiation 100 mW/cm^2, 30 seconds; Materials and methods-singlet oxygen detection: 100 mW/cm^2, 30 min?

Re: We are sorry for the error! This error has been corrected in Materials and methods – singlet oxygen detection.

7. Figure 6: lack of the light control and dark control. See also the point 1.

Re: Thank you for the advice! As shown in **Figure R3**, there was no obvious killing ability of MCC and OxgeMCC-r SAE for the tested concentrations in the dark condition. The light control with NIR only also showed a negligible effect to the viability. **Figure R3** has been

added in the revised supporting file as **Supplementary Fig. 15**, and corresponding descriptions have been added in the revised manuscript **(Page 8, paragraph 2**).

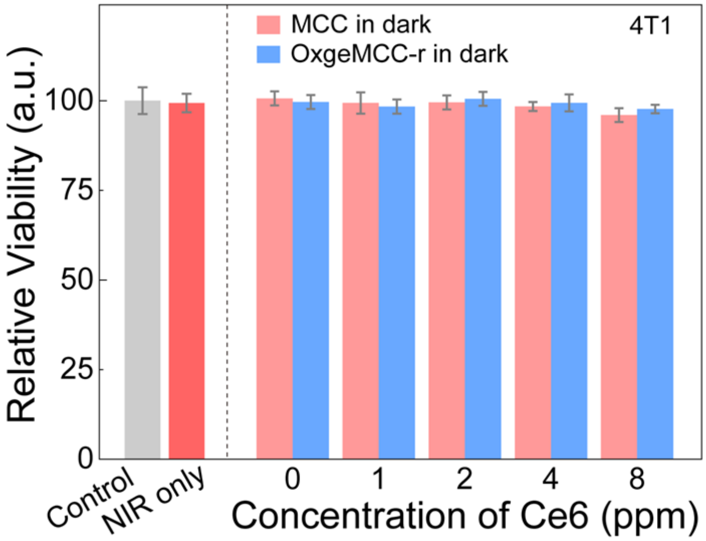

Figure R3. **4T1 cell viability under dark condition**. 4T1 cell viability assay of control group, NIR group (671 nm light irradiation), MCC in dark, and OxgeMCC-r SAE in dark of different concentrations. (The concentration is based on Ce6; 671 nm laser power density: 100 mW cm^{-2}; irradiation time: 30 seconds).

8. What is the subcellular localization of MCC-r and OxgeMCC-r?

Re: Thank you for the advice! The Ce6 fluorescence within cells incubated with both MCC and OxgeMCC-r SAE showed accurate overlap with the signals of LysoTracker after 4 h incubation (**Figure R10**), suggesting subcellular localization of both MCC and OxgeMCC-r SAE is in endosomes/lysosomes. **Figure R10** has been added in the revised supporting file as **Supplementary Fig. 14**, and corresponding descriptions have been added in the revised manuscript **(Page 8, paragraph 2**).

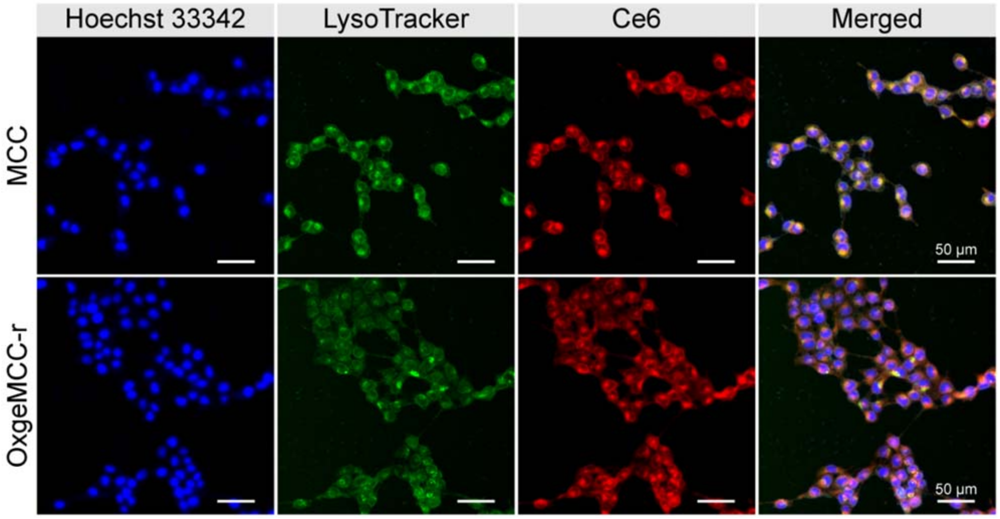

Figure R10. **Subcellular localization of MCC and OxgeMCC-r SAE**. Confocal images of MCC-r and OxgeMCC-r SAEs incubated with 4T1 cells for 4 h. Blue, red, and green colors represent Hoechst 33342, LysoTracker-stained endosomes/lysosomes, and Ce6 fluorescence, respectively.

9. For more than two samples ANOVA (e.g. Fig. 5c/6a/6b/8c) or ANCOVA (Fig. 8a) needs to be used to analyse the statistical significance.

Re: Thank you! Added as suggested. Asterisk (*) denotes statistical significance between bars (*$p < 0.05$, **$p < 0.01$, ***$p < 0.001$) conducted using GraphPad Prism 6.0.

REVIEWERS' COMMENTS:

Reviewer #1 (Remarks to the Author):

I think this work after careful revision is now acceptable for publication.

Reviewer #2 (Remarks to the Author):

I would like to thank the authors for their detailed responses and additional experiments. The manuscript is now much improved and is suitable for publication.

Reviewer #3 (Remarks to the Author):

The authors have addressed all the comments of the reviewers and improved the article substantially. Therefore, it can be accepted for publication.

Good luck!

Mateusz Kwitniewski

Point-by-point response to reviewers' comments

We thank all the reviewers for recommending the acceptance of our revised manuscript.

Reviewer #1 (Remarks to the Author):

Reviewer(s)' Comments to Author:

Comments: *I think this work after careful revision is now acceptable for publication.*

Re: Thanks very much for your recommendation of our work.

Reviewer #2 (Remarks to the Author):

Reviewer(s)' Comments to Author:

Comments: *I would like to thank the authors for their detailed responses and additional experiments. The manuscript is now much improved and is suitable for publication.*

Re: Thanks very much for your recommendation of our work.

Reviewer #3 (Remarks to the Author):

Reviewer(s)' Comments to Author:

Comments: *The authors have addressed all the comments of the reviewers and improved the article substantially. Therefore, it can be accepted for publication.*

Good luck!

Mateusz Kwitniewski

Re: Thanks very much for your recommendation of our work.
